# Preparation, structure elucidation, and antioxidant activity of new bis(thiosemicarbazone) derivatives

**DOI:** 10.3906/kim-2002-76

**Published:** 2020-08-18

**Authors:** Hasan YAKAN

**Affiliations:** 1 Department of Mathematics and Science Education, Faculty of Education, Ondokuz Mayis University, Samsun Turkey

**Keywords:** Thiosemicarbazides, Schiff bases, DPPH, structure–activity relationship, spectroscopic methods

## Abstract

Schiff-base–bearing new bis(thiosemicarbazone) derivatives were prepared from terephthalaldehyde and various thiosemicarbazides. FT–IR, ^1^H NMR, ^13^C NMR, and UV–Vis spectroscopic methods and elemental analysis were used to elucidate the identification of the synthesized molecules. The in vitro antioxidant activity of the synthesized compounds was analysed with the 1,1-diphenyl-2-picryl hydrazyl free-radical–trapping process. The synthesized compounds exhibited lower antioxidant activity than the standard ascorbic acid. IC_50_ values of the synthesized molecules measured from 3.81 ± 0.01 to 29.05 ± 0.11 μM. Among the synthesized compounds, compound **3** had the best antioxidant activity. Moreover, this study explained the structure–activity relationship of the synthesized molecules with different substituents in radical trapping reactions.

## 1. Introduction

Thiosemicarbazones are an important class of organic chemicals which have an -NH-C(=S)NH-N= bond. They are used as versatile intermediates for synthesizing numerous moieties as well. Thiosemicarbazones have shown numerous medicinal properties and biological activities such as anticonvulsant [1], antituberculosis [2], urease inhibitory [3], anticancer [4], antiviral [5], antimicrobial [1], antibacterial [6], and antioxidant activity [7–11].

Schiff bases have an extensive range of biological and medicinal properties, such as antimicrobial [12], antiinflammatory [13], antibacterial [14], antifungal [15], insecticidal [16], anticancer [17] antituberculosis [18], anticonvulsant [19], and antioxidant [20] activity. They are also used as pigments, catalysts, polymer stabilisers, and intermediates in organic synthesis [21].

The significance of free radicals and reactive oxygen species (ROS) in the pathogenicity of various diseases such as metabolic disorders, reperfusion damage, inflammatory diseases, cellular aging, and cancer has attracted substantial consideration [22–24]. Many of these illnesses occur with the bulking of free radicals in humans. Hence, antioxidants have been noted to play a major role in preserving humans from many potentially fatal illnesses.

In view of this evidence, Schiff bases are important not only in possessing biological activities, but also in pharmacological chemistry. In this paper, a series of novel bis(thiosemicarbazones) based on Schiff bases were prepared by condensation reaction with terephthalaldehyde and various thiosemicarbazides under reflux in ethanol. The structures of the new compounds were characterized by Fourier-transform infrared (FT–IR), proton–carbon nuclear magnetic resonance (1 H NMR–13 C NMR), and ultraviolet–visible (UV–Vis) spectroscopic techniques, as well as elemental analysis. Antioxidants are very important reagents because they can trap free radicals and reduce their damaging effects on our bodies. Therefore, the in vitro antioxidant activity of all of the obtained molecules were evaluated by the 1,1-diphenyl-2-picryl hydrazyl (DPPH) freeradical trapping process. This paper investigated the effects of diverse structural features on the antioxidative activity of all the compounds. Moreover, I studied the electronic effect of electron-donating and electronwithdrawing groups as the substituents on antioxidant activity, as well as their positions. The aim of this study was to explain the structure–activity relationship of the products with various substituents in radical scavenging reactions.

## 2. Experimental

### 2.1. Measurement and reagents

All chemicals and solvents were purchased from Merck & Co., Inc. (Kenilworth, NJ, USA) or Sigma-Aldrich Chemical Company (St. Louis, MO, USA). They were used without further purification. A Stuart SMP30 melting point apparatus (Cole-Parmer Instruments Co., Vernon Hills, IL, USA) was used to determine melting points. Infrared (IR) spectra were recorded with a Bruker Alpha Fourier transform IR (FT–IR) spectrometer (Bruker BioSpin Corp., Billerica, MA, USA). NMR spectra were taken on a Bruker Avance DPX–400 (400 MHz) spectrophotometer using dimethyl sulfoxide- *d_6_* (DMSO– *d_6_* ) as solvent. A Eurovector EA3000 elemental analyser was used for elemental analysis (Eurovector S.r.l., Pavia, Italy). UV–Vis spectra and absorption data were obtained with a Shimadzu UVM-1240 UV–Visible spectrophotometer (Shimadzu Corporation, Kyoto, Japan).

### 2.2. Synthesis of bis(thiosemicarbazones) based on Schiff base (1–10)

**Scheme 1:**
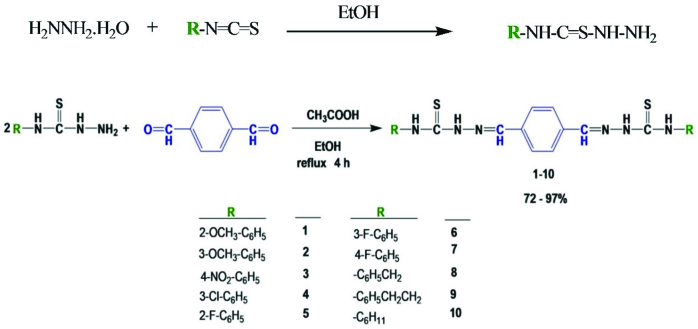
Synthetic route for bis(thiosemicarbazones) based on Schiff base (1–10).

To a solution of hydrazine monohydrate (5.0 mmol) in ethanol (10 mL), a suspension of an appropriate isothiocyanate (5.0 mmol) in ethanol (10 mL) was added dropwise with vigorous stirring and cooling in an ice bath. The mixture was allowed to stand overnight. The solid molecule so formed was filtered and dried, producing thiosemicarbazides as an intermediate product. Terephthalaldehyde (2.0 mmol) and thiosemicarbazide derivatives (4.0 mmol) were stirred into 50 mL of absolute ethanol in the presence of 3 drops of glacial CH_3_COOH. The reaction mixture was refluxed for 4 h. When the reaction was finished, the reaction mixture was cooled to room temperature. The solid products so formed were filtered and dried (Scheme 1). They were obtained according to an earlier procedure, with slight changes [25].

### 2.3. Antioxidant activity

The antioxidant activities of the newly synthesized compounds were evaluated based on the studies of Brand-Williams (with slight modification) using DPPH radical scavenging activity analysis [26]. Stock solutions of the compounds in DMSO at a concentration of 250 μM were prepared. The previously prepared DPPH solution was used in 3 mL with a concentration of 100 μM in ethanol. DPPH solution was added to different concentrations of the compound solutions (0.5–10.0 μM) and enough ethanol to bring the volume up to a total of 5 mL. This mixture was allowed to stand at room temperature for 30 min in the dark. The measurement against the gap was taken at 517 nm, and the radical scavenging activity was expressed as a percentage of inhibition. Percentage of inhibition was calculated as follows [27]:

Inhibition (%) = [(A_0_ – A_1_) / A_0_ × 100];

where A_0_ is the absorbance of the control and A1 is the absorbance of the sample. The inhibition curves were prepared, and the half-maximal inhibitory concentration (IC_50_) values were calculated using linear regression analysis [27].

## 3. Results and discussion

### 3.1. Physical properties

The results for the physical properties, yields, melting points, and elemental analyses are given in Tables 1 and 2.

**Table 1:** Physical properties of the synthesized compounds.

Comp.	Compound name	-R	Yield %	Melting point (◦C)	Colour
**1**	(2,2')-1,4-Benzenedicarboxaldehyde-1-methanylylidene-bis(4-(2-methoxyphenyl)thiosemicarbazone)	2-OCH_3_-C_6_H_5_	86	>300	Yellow
**2**	(2,2')-1,4-Benzenedicarboxaldehyde-1-methanylylidene-bis(4-(3-methoxyphenyl)thiosemicarbazone)	3-OCH_3_-C_6_H_5_	95	222–224	Yellow
**3**	(2,2')-1,4-Benzenedicarboxaldehyde-1-methanylylidene-bis(4-(4-nitrophenyl)thiosemicarbazone)	4-NO_2_-C_6_H_5_	86	270–273	Light orange
**4**	(2,2')-1,4-Benzenedicarboxaldehyde-1-methanylylidene-bis(4-(3-chlorophenyl)thiosemicarbazone)	3-Cl-C_6_H_5_	90	>300	Dark yellow
**5**	(2,2')-1,4-Benzenedicarboxaldehyde-1-methanylylidene-bis(4-(2-fluorophenyl)thiosemicarbazone)	2-F-C_6_H_5_	72	>300	Yellow
**6**	(2,2')-1,4-Benzenedicarboxaldehyde-1-methanylylidene-bis(4-(3-fluorophenyl)thiosemicarbazone)	3-F-C_6_H_5_	97	>300	Dark yellow
**7**	(2,2')-1,4-Benzenedicarboxaldehyde-1-methanylylidene-bis(4-(4-fluorophenyl)thiosemicarbazone)	4-F-C_6_H_5_	91	268–270	Yellow
**8**	(2,2')-1,4-Benzenedicarboxaldehyde-1-methanylylidene-bis(4-(benzyl)thiosemicarbazone)	C6H5CH2	81	>300	Yellow
**9**	(2,2')-1,4-Benzenedicarboxaldehyde-1-methanylylidene-bis(4-(2-phenylethane)thiosemicarbazone)	C_6_H_5_CH_2_CH_2_	80	280–283	Yellow
**10**	(2,2')-1,4-Benzenedicarboxaldehyde-1-methanylylidene-bis(4-(cyclohexzyl)thiosemicarbazone)	C_6_H_11_	90	>300	Light orange

**Table 2:** Elemental analysis results of the compounds.

Comp.	Molecular mass (g/mol)	Molecular formula	Calculated			Experimental		
			C%	H%	N%	(C)%	(H)%	(N)%
**1**	492.14	C_24_H_24_N_6_O_2_S_2_	58.52	4.91	17.06	58.69	4.87	16.98
**2**	492.14	C_24_H_24_N_6_O_2_S_2_	58.52	4.91	17.06	58.32	4.95	17.16
**3**	522.09	C_22_H_18_N_8_O_4_S_2_	50.57	3.47	21.44	50.80	3.38	21.35
**4**	500.04	C_22_H_18_Cl_2_N_6_S_2_	52.69	3.62	16.76	52.55	3.66	16.59
**5**	468.10	C_22_H_18_F_2_N_6_S_2_	56.39	3.87	17.94	56.21	3.83	17.91
**6**	468.10	C_22_H_18_F_2_N_6_S_2_	56.39	3.87	17.94	56.24	3.84	17.90
**7**	468.10	C_22_H_18_F_2_N_6_S_2_	56.39	3.87	17.94	56.55	3.79	17.86
**8**	460.15	C_24_H_24_N_6_S_2_	62.58	5.25	18.25	62.33	5.17	18.37
**9**	488.18	C_26_H_28_N_6_S_2_	63.90	5.78	17.20	63.72	5.92	17.11
**10**	444.21	C_22_H_32_N_6_S_2_	59.42	7.25	18.90	59.65	7.11	19.01

### 3.2. Vibrational frequencies

In the FT-IR spectra, the signal of the aldehyde group (-CHO, 2 bands) of the starting material was not observed at 2780–2660 cm^-1^. Moreover, the symmetric and asymmetric stretching peaks of the amino group (-NH_2_) did not show at 3520–3250 cm^-1^. Instead, new vibrations for the –C=N stretching peaks of imine group were observed at 1599–1522 cm^-1^. These results indicated a successful reaction, as expected. For the synthesized compounds (1–10), the amine group (–NH) vibration peaks were observed at 3368–3269 cm^-1^, the –C=S signals of the thiosemicarbazone region were observed at 1462–1409 cm^-1^, and the -C–N group vibrations were observed at 1222–1152 cm^-1^. The other considerable vibrations were in the spectrum of compounds **1-10** resulting from the –C-O, –NO_2_, Ar–Cl, Ar–F, and –CH_2_, functions, respectively. The -C–O stretching vibrations of compounds **1** and **2** were observed at 1045 and 1048 cm^-1^. The nitro group (–NO_2_) stretching peaks were observed at around 1508 and 1317 cm^-1^ for compound **3** . The Ar–Cl vibration signal of compound 4 was observed at 767 cm^-1^. For compounds **5-7** , Ar–F vibration signals were observed at 940, 932, and 936 cm^-1^, respectively. In the literature, Ar–Cl and Ar–F vibration peaks were observed at around 811–856 cm^-1^, 900–1038 cm^-1^, respectively [10,28]. 

**Figure 1:**
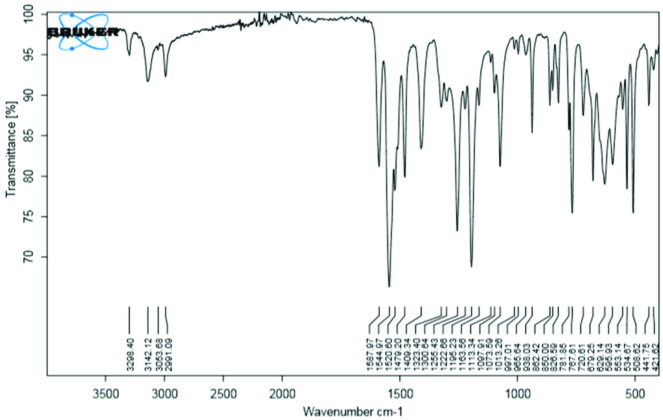
FT-IR spectrum of compound 4.

In compound 4, the –NH stretching vibration was observed at 3298 cm^-1^. The –C=N vibration signal appeared at 1544 cm^-1^, the –C=S signal of thiosemicarbazone region was observed at 1409 cm^-1^, and the -C–N stretching vibration appeared at 1195 cm^-1^, as shown in Figure 1. The IR peaks of the compounds are presented in Table 3 (see Supplementary information). The frequency data of all of the molecules were consistent with those reported for similar compounds [8,10,28,29].

**Table 3:** Experimental FT-IR values of the compounds (cm^-1^).

Comp.	-NH	Ar.CH	Aliph.CH	C=N	C=S	C-N	Spec. vib.
**1**	3269	3122	2969	1599	1462	1192	C–O:1045
**2**	3307	3122	2940	1545	1455	1217	C–O:1048
**3**	3306	3157	-	1537	1415	1186	NO_2_:1508–1317
**4**	3298	3142	-	1544	1409	1195	C–Cl:767
**5**	3331	3132	-	1555	1456	1190	C–F:940
**6**	3335	3144	-	1593	1414	1161	C–F:932
**7**	3306	3134	-	1538	1409	1152	C–F:936
**8**	3308	3163	2933	1540	1415	1222	-
**9**	3368	3158	2923	1522	1451	1182	-
**10**	3292	3164	2928	1525	1446	1153	-

### 3.3. ^1^H NMR interpretations

The ^1^H NMR spectra of all of the compounds were taken in DMSO- *d_6_* ; the chemical shifts are given in Table 4. Signals of DMSO- *d_6_* and water in DMSO (HOD, H_2_O) were seen around 2.00, 2.55 (quintet), and 3.40 (variable, depend on the solvent and its concentration) ppm, respectively [30,31].

**Table 4:** ^1^H NMR (δ , ppm, in DMSO- *d_6_* ) values of synthesized compounds.

Comp.	H1	H2	H3	H4	H5	H6	Ar-NH	NH-N	CH=N	Spec. peaks
**1**	-	8.22–8.21 d	7.23–7.19 td	7.00–6.96 td	7.13–7.10 d	7.89 s	10.04 s	12.02 s	8.19 s	-OCH_3_:3.90, s
**2**	7.16-7.14 d	-	7.25–7.21 t		6.76–6.73 dd	7.91 s	10.07 s	11.86 s	8.14 s	-OCH_3_:3.72, s
**3**	6.61–6.59 d	8.28–8.25 d	-	8.28–8.25 d	6.61–6.59 d	8.07 s	10.50 s	12.30 s	8.00 s	-
**4**	7.79 s	-	7.62–7.60 dd	7.43–7.39 t	7.29–7.26 dd	7.97 s	10.24 s	12.05 s	8.21 s	-
**5**	-	7.56–7.52 m	7.35–7.22 m			7.94 s	11.04 s	12.06 s	8.18 s	-
**6**	7.58–7.54 d	-	7.43–7.33 m		7.02–6.98 t	7.92 s	10.18 s	11.97 s	8.15 s	-
**7**	7.22–7.17 d	7.60-7.55 d	-	7.60–7.55 d	7.22–7.17 d	7.96 s	10.18 s	11.94 s	8.18 s	-
**8**	7.21–7.17, t	7.32–7.26 m			7.21–7.17, t	7.80 s	9.10–9.07 t	11.63 s	8.05 s	-CH_2_:4.82–4.80d
**9**	7.26–7.22 m	7.36–7.29 m			7.26–7.22 m	7.82 s	8.63–8.60 t	11.62 s	8.08 s	Ar-CH_2_: 2.97– 2.93, t -CH_2_-N: 3.82– 3.76, q
**10**	-	-	-	-	-	7.82 s	8.07 s	11.50 s	8.05 s	-CH: 4.24– 4.18, p -CH_2_: 1.91– 1.14, m

**Figure 2:**
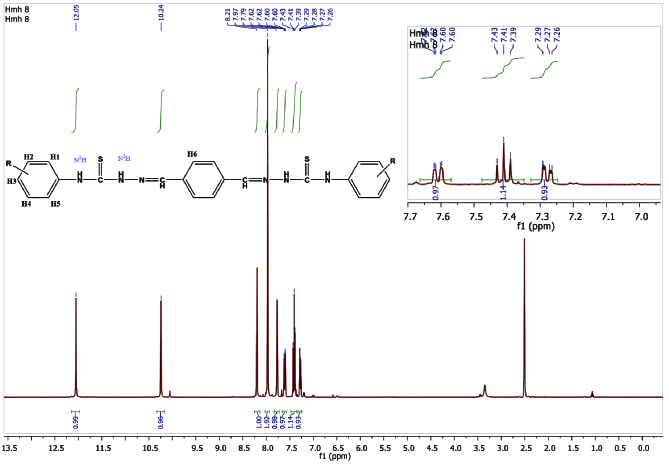
^1^H NMR spectrum of compound **4** .

In compound 4, the signal of imine (-CH=N) was observed as a singlet at 8.21 ppm. The -N^1^H and -N^2^H proton signals of the thiosemicarbazone region were observed as a singlet at 10.24 and 12.05 ppm, respectively. The aromatic proton signals of the isatin ring (H1–H5) were detected between 7.79 and 7.26 ppm (Figure 2). The H1 proton showed as a singlet peak at 7.79 ppm. The H3 proton coupled to the H4 and H5 protons and observed doublet of doublets peakes at 7.62–7.60 ( *J* = 7.4, 1.8 Hz) ppm. The H4 proton coupled to the H3 and H5 protons and showed triplet peaks at 7.43–7.39 ( *J* = 8.1 Hz) ppm. The H5 proton coupled to the H3 and H4 protons and showed doublet of doublets peaks at 7.29–7.26 ( *J* = 8.0, 3.0 Hz) ppm. The H6 proton was detected as a singlet peak at 7.97 (s, 2H) ppm. For compounds **1** and **2** , the proton signals of the methoxy group (–OCH_3_) showed as a singlet at 3.90 and 3.72 ppm. For compound 8, the proton signal of methylene group (–CH_2_) was observed as a doublet at 4.82–4.80 (2H, d, *J* = 6.1 Hz) ppm; the –NH proton signal was detected as a triplet at 9.10–9.07 (1H, t, *J* = 6.1 Hz). Similarly, the methylene group (-CH ^2^ –N) proton signal was observed as a quartet at 3.82–3.76 (2H, q, *J* = 5.8, 2.4 Hz) ppm; the methylene group (Ar–CH_2_) proton signal showed as a triplet at 2.97–2.93 (2H, t, *J* = 2.4 Hz) ppm. The –NH proton signal was detected as a triplet at 8.63–8.60 (1H, t, *J* = 5.8 Hz) for compound **9** (see Supplementary information). These results are highly consistent with values for similar compounds. [7,10,25,28].

### 3.4. ^13^C NMR interpretations

The ^13^C NMR spectra of the compounds were taken in DMSO- *d_6_* ; the chemical shifts are summarized in Table 5. In the compounds ( **1-9** ), the aromatic C signals of the aryl ring (C1–C6) were observed between 163.4 and 111.4 ppm and those from the terephthalaldehyde ring (C9–C10) were observed between 141.1 and 128.2 ppm. The characteristic -C=N (C8) peaks of imine were observed at 144.1 and 141.0 ppm for all compounds (1–10). The other remarkable characteristic, -C=S (C7) peaks of the thiosemicarbazone region, were observed at 178.1 and 175.6 ppm for all compounds ( **1-10** ). In compounds **1** and **2** , the methoxy carbon atoms (–OCH_3_) resonated at 56.4 and 56.1 ppm. For compound **8** , the methylene carbon signal was detected at 47.1 ppm. In compound **9** , the methylene carbon signals (Ar-CH_2_ and -CH_2_–N) were detected at 35.3 and 45.5 ppm. For compound **10** , the aliphatic C signals of the cyclic ring (C1–C6) were observed between 53.1 and 25.4 ppm. Furthermore, in compounds **5** , **6** , and **7** , the C atoms (for C1–C6) were also split into doublets caused by interacting with the atomic nucleus of F. Additionally, in compounds **1-7** , the signals of the carbon were shifted downfield (high values of δ) relative to the signal of benzene (128.5 ppm) due to the presence of the 2–OCH_3_ (152.1 ppm), 3–OCH_3_ (159.8 ppm), 4–NO_2_ (145.9 ppm), 3–Cl (143.2 ppm), 2–F (159.0 ppm), 3–F (163.4 ppm), and 4–F (161.4 ppm) atoms/groups (see Supplementary information).

**Table 5:** ^13^C NMR data of compounds 1–10 (δ /ppm).

Comp.	C1	C2	C3	C4	C5	C6	C7	C8	C9	C10	R
**1**	152.1	111.8	124.6	120.4	128.1	126.4	175.6	142.2	135.9	128.2	-OCH3: 56.4
**2**	111.4	159.8	112.3	129.4	118.6	136.0	176.3	142.9	140.7	128.5	-OCH3: 56.1
**3**	125.1	124.2	145.9	124.2	125.1	127.0	176.0	144.1	135.9	128.5	-
**4**	125.7	143.2	125.5	130.1	124.8	132.6	176.4	141.0	135.9	128.4	
**5**	159.0 156.6	116.3 116.1	127.7 127.6	124.5 124.4	130.7 130.2	128.7 128.6	177.8	142.9	135.9	128.3	-
**6**	113.0 112.8	163.4 160.7	112.5 112.3	130.1 130.0	122.0 121.9	141.4 141.2	176.5	143.2	135.9	128.4	-
**7**	128.8 128.7	115.3 115.1	161.4 158.9	115.3 115.1	128.8 128.7	135.9 135.8	176.9	142.8	141.1	128.3	-
**8**	127.3	128.0	127.3	128.0	127.3	139.9	178.1	142.1	135.9	128.7	-CH2: 47.1
**9**	127.9	128.9	126.7	128.9	127.9	139.7	177.5	141.8	135.9	129.1	Ar-CH2: 35.3 -CH2-N: 45.5
**10**	32.2	25.4	25.6	25.4	32.2	53.1	176.2	141.9	135.8	127.9	-

**Figure 3:**
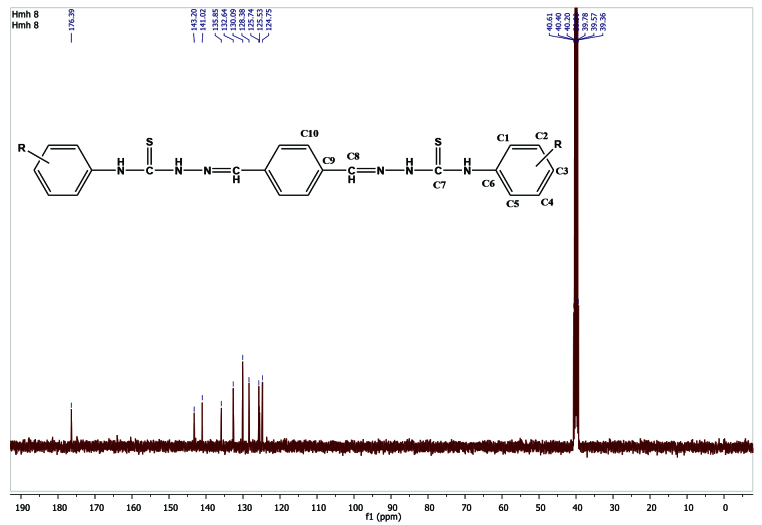
^13^C NMR spectrum of compound 4.

The ^13^C NMR spectrum of compound 4 had 10 different resonances, consistent with the target structure shown in Figure 3.

In compound 4, the -C=S (C7) signal of the thiosemicarbazone region was observed at 176.4 ppm. The characteristic -C=N (C8) peak of imine was observed at 141.0 ppm. The carbons (C1–C6) of aryl the ring were detected at 143.2, 132.6, 130.1, 125.7, 125.5, and 124.8 ppm, respectively. The aromatic C signals of the terephthalaldehyde region (C9–C10) were observed at 135.9 and 128.4 ppm. The C2 (143.2 ppm) carbon atoms shifted downfield (high values of δ) , which was caused by the presence of the chlorine group. These results are in agreement with the data reported for similar compounds [8–10,28,29].

### 3.5. UV–Vis interpretations

The electronic absorption spectra of the new bis(thiosemicarbazones) based on Schiff base derivatives (^1-10^) show that in all obtained products absorption bands appeared in the range of 256–406 nm in dimethylsulfoxide (see Figures S28–S36). The electronic spectra of these compounds show remarkable absorption bands of the aromatic ring (C=C) and thiosemicarbazone (C=S)–imine (CH=N) region, owing to transitions of a π → π* and an n → π*. The π → π*-type transitions of C=C on the aromatic ring are seen at 256 nm, except in compound 1 (258 nm). This transition is a characteristic benzenoid (B) band of the aromatic structure [32]. While the n → π* transitions of the imine C=N double bond were seen at 282–296 nm for all compounds, the bands at 352–406 nm are assigned to the electronic transition n → π* of the thiosemicarbazone moiety (C=S).

**Figure 4:**
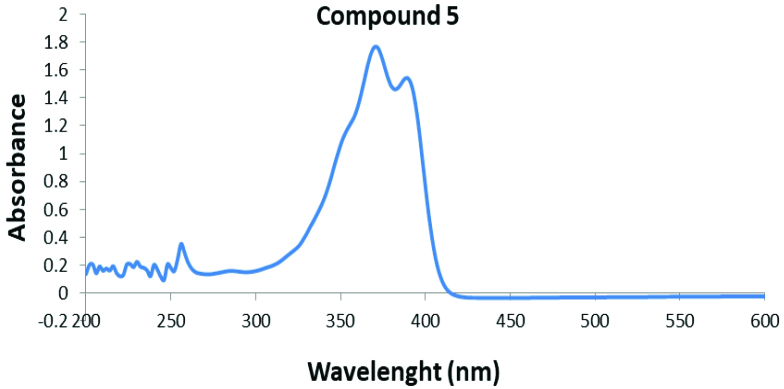
UV–Vis spectrum of compound 5 in DMSO.

In compound 5, the 3 n → π* transitions of the thiosemicarbazone moiety (C=S) are at 352 (shoulder), 370, and 390 nm, respectively, as shown in Figure 4. While the bands in the spectral region 286 nm (shoulder) can be assigned to the azomethine C=N double bond to the n → π* transitions, the π → π* transitions of C=C on the aromatic structure are seen at 256 nm, as shown in Figure 4. The electronic spectral data of all of the compounds are summarized in Table 6. These values are in agreement with the results that were reported for similar compounds [11,33–35].

**Table 6:** Electronic spectral data (nm) of all the compounds (1–10) in DMSO.

Compound	π → π*, B band	n → π*, C=N	n → π*, C=S
**1**	258	296	360, 380, 396
**2**	256	296	358, 378, 396
**3**	256	290	356, 372, 392
**4**	256	284	364, 384, 406
**5**	256	286	352, 370, 390
**6**	256	282	356, 372, 394
**7**	256	288	354, 370, 388
**8**	256	292	358, 378, 396
**9**	256	294	358, 376, 396
**10**	256	286	352, 370, 388

### 3.6. Evaluation of antioxidant activity

**Figure 5:**
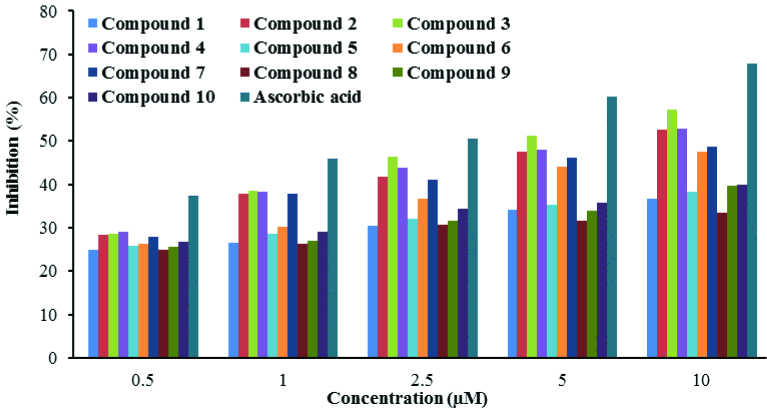
Percentage of inhibition calculated by the DPPH method for ascorbic acid and compounds 1–10.

Antioxidant activity of all of the molecules was determined according to a reported procedure [26,27] as described in the experimental literature, with minor modifications. It can be assumed that the hydrogen losing capacities/abilities or structurally stable radical creation after the interaction of the compounds with DPPH free radicals is related to the antioxidant activity of the molecule being studied [36]. Percentage inhibition changes of ascorbic acid and synthesized molecules are given in Figure 5. All compounds followed a steadily increasing sequence depending on the concentration. The synthesized molecules produced lower free radical scavenging at all concentration ranges compared to ascorbic acid.

The antioxidant activity of all sample molecules is expressed with IC50 values in the results given in Table 7. IC_50_ values of the molecules were found to be between 3.81 ± 0.01 and 29.05 ± 0.11 μM. The DPPH test is thought to cause hydrogen atom transfer reaction, which causes formation of the novel radical [37]. Hence, antioxidant compounds can react with DPPH free radicals by giving hydrogen atoms or by electron donation via a free radical attack on these compounds [38,39]. According to this reaction (DPPH• + R-NH → DPPH-H + R-N•) , weaker hydrogen bonds are necessary for higher antioxidant activity in a compound. The strength of these hydrogen bonds (–NH group in the thiosemicarbazone moiety) is proportional to electron density. Consequently, antioxidant activity depends on two things: the first being the capacity/skill of compounds to lose hydrogen atoms, and the second being the stability of the formed radical [36,39,40]. Therefore, the structure of the tested compounds and electronic effects of groups/substituents in structures plays a significant role in antioxidant activity [36,37,41]. It is generally known that there are 3 types of electron effects: mesomeric (or resonance), inductive, and steric hindrance. Substituents on the compounds, which are known as electrondonating groups (OH, NH_2_, OCH_3_, CH_3_, etc.) and electron-withdrawing groups (NO2 , CN, CF_3_, F, Cl, Br, etc.), significantly affect antioxidant activity [42]. On the other hand, the position of these groups/substituents in the molecules plays a major role in antioxidant activity as well.

**Table 7:** IC_50_ values for the synthesized compounds **1-10** .

Compounds	DPPH scavenging activity, IC_50_ (μM)*
**1**	9.29 ± 0.01
**2**	7.72 ± 0.04
**3**	3.81 ± 0.01
**4**	7.48 ± 0.04
**5**	8.69 ± 0.04
**6**	9.92 ± 0.05
**7**	9.28 ± 0.05
**8**	29.05 ± 0.11
**9**	8.26 ± 0.04
**10**	16.96 ± 0.09
Ascorbic acid	2.99 ± 0.01

*IC_50_ = Concentration (μM) exhibiting 50% inhibition of DPPH radical. Values are expressed as means (n = 3).

In view of this information, compound **3** , possessing a nitro group ( *p* –NO_2_) which had strong electronwithdrawing through inductive effect, showed the strongest antioxidant activity against the DPPH radical amongst all test molecules. The reason is that the electron-withdrawing groups reduce the bond strength between nitrogen and hydrogen atoms, thus decreasing electron density in the structure, which causes easier loss of the hydrogen atom. In a previous study, compounds containing nitro groups, which draw electrons on the ring, showed much higher antioxidant activity than various substituted structures [10]. At the same time, experimental results on halogens showed different activities depending on their position according to the –NH. Antioxidant activity order was **4** ( *m* –Cl) > **5** ( *o* –F) > **7** ( *p* –F) > **6** ( *m* –F). In similar studies, antioxidant activity among the halogen-containing compounds was –Cl > –F [8,43,44]. As for the effect of each position of the fluorine atom, *o* –F and *p* –F substituents have a strong inductive electron-attracting effect which causes easier hydrogen atom loss, whereas *m* –F substituent has poor inductive effect.

Compound **2** ( *m* –OCH_3_) had higher antioxidant activity than compound **1** ( *o* –OCH_3_). While the *m* –OCH_3_group is an electron-withdrawing substituent with negative inductive effect, the *o* –OCH_3_ group is an electron-donating substituent with a positive resonance effect. The *m* –OCH_3_ substituent provides easier loss of the hydrogen atom via decreased strength of the N-H bond, whereas the *o* –OCH_3_ group has the opposite effect [45]. Compound **10** (cyclic ring) has higher antioxidant activity than compound **8** (benzyl group). Compound **8** had difficulty losing the hydrogen atom because the benzyl group is a more electron-donating substituent than that of the cyclic ring. Consequently, IC_50_ values for the products followed the order of **3** > **4** > **2** > **9** > **5** > **7** > **1** > **6** > **10** > **8** .

## 4. Conclusion

In this study, Schiff-base–bearing new bis(thiosemicarbazones) have been obtained, with excellent yields of 72%–97%. The structure of all of the compounds was elucidated using spectroscopic methods such as FT–IR, ^1^H NMR, ^13^C NMR, and UV–Vis, as well as elemental analysis. The in vitro antioxidant activity of the synthesized molecules was determined using the DPPH free-radical trapping process. IC_50_ values of the newly obtained molecules ranged from 3.81 ± 0.01 to 29.05 ± 0.11 μM. Among the synthesized compounds, compound **3** had the best satisfactory antioxidant activity against the DPPH radical. Additionally, the structure–activity relationships were studied in relation to the presence, types, and position of substituents. Among all of the test molecules, a compound which possesses electron-attracting groups has higher antioxidant activity against DPPH free radicals than electron-donating groups.

Supplementary Materials
