# Supplementary material for: Preparation, structure elucidation, and antioxidant activity of new bis(thiosemicarbazone) derivatives
Source: Turk J Chem. 2020 Aug 18;44(4):1085–99. doi: 10.3906/kim-2002-76 (PMC7751920; doi:10.3906/kim-2002-76)
Supplement: Supplementary file 1 — Supplementary Materials [file turkjchem-44-1085-sup001.pdf]

Supplementary materials

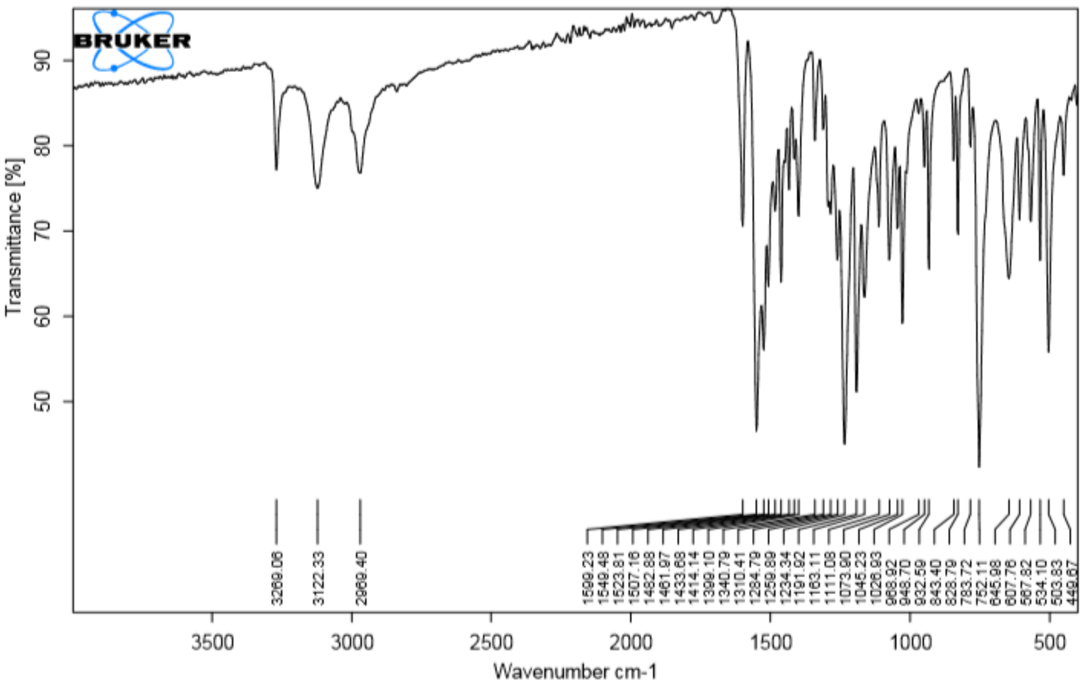

Figure S1. IR spectrum of compound **1**.

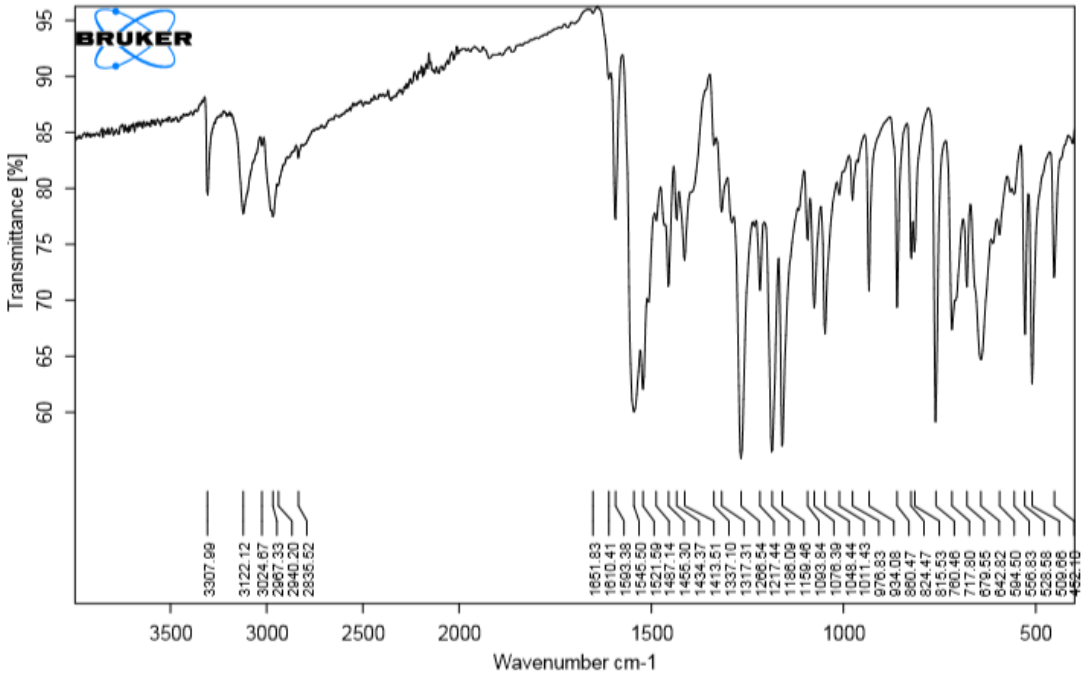

Figure S2. IR spectrum of compound **2**.

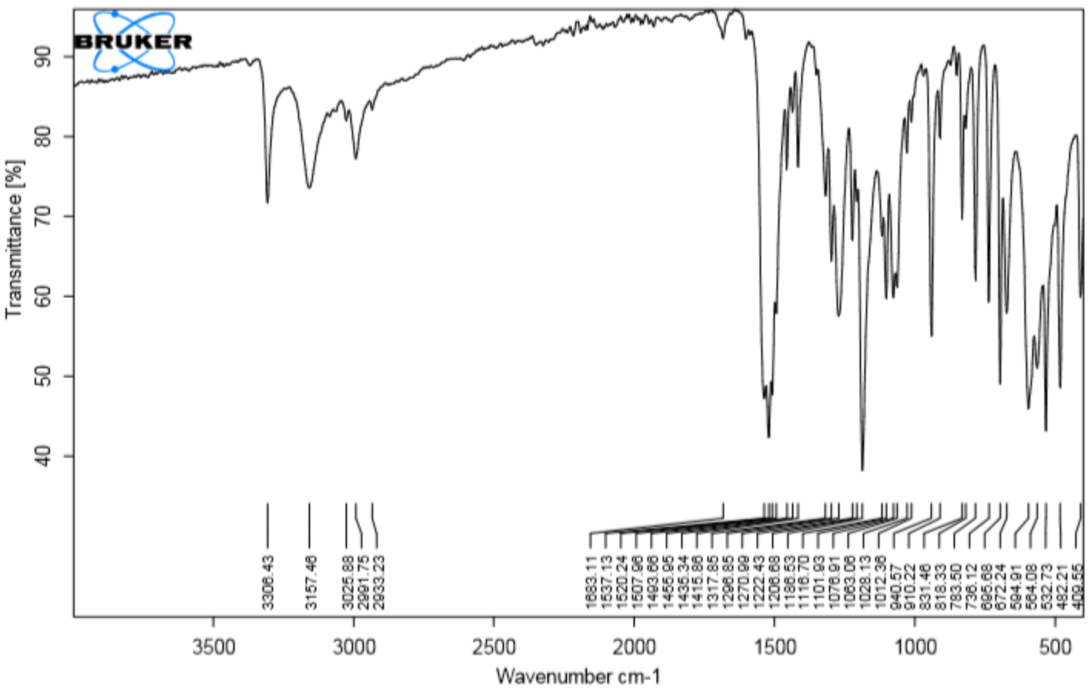

Figure S3. IR spectrum of compound **3**.

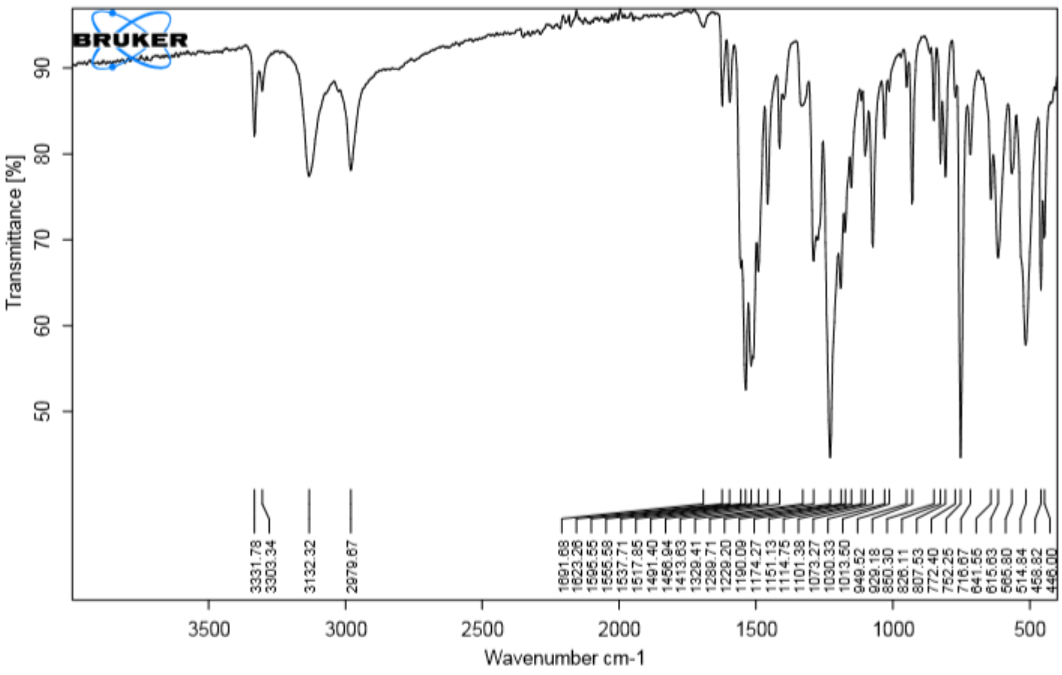

Figure S4. IR spectrum of compound **5**.

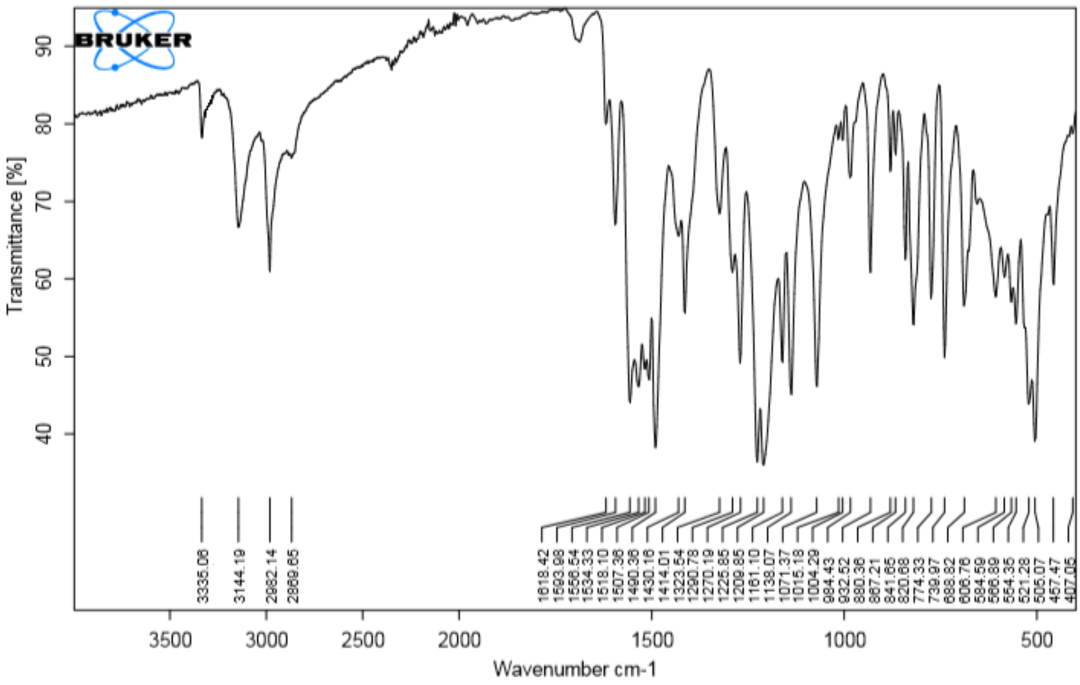

Figure S5. IR spectrum of compound **6**.

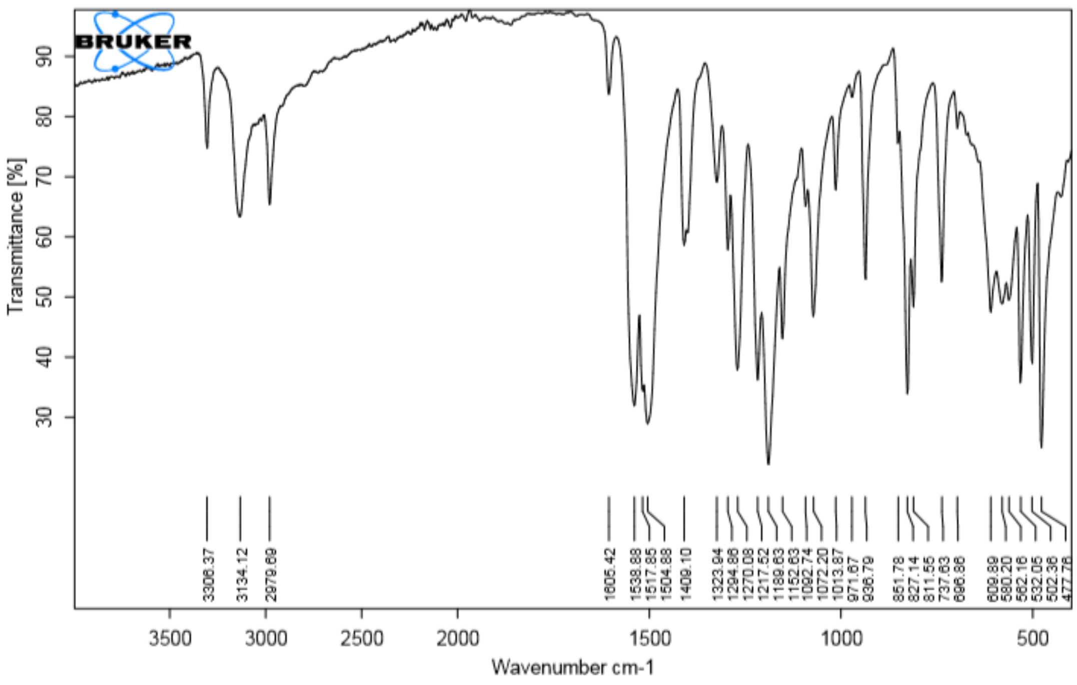

Figure S6. IR spectrum of compound **7**.

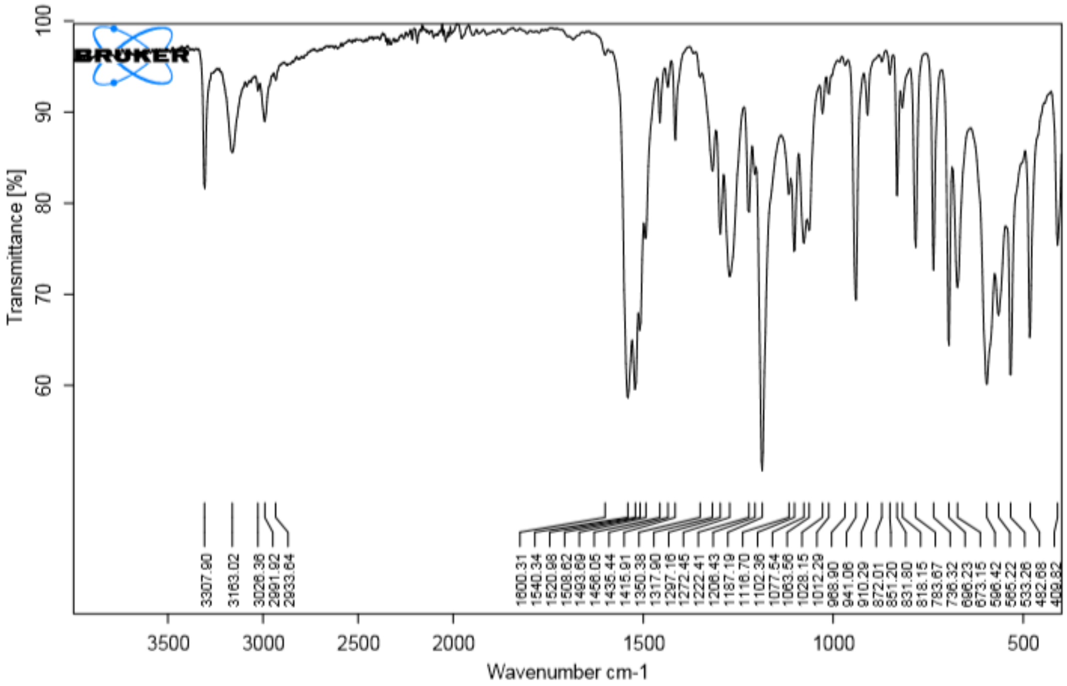

Figure S7. IR spectrum of compound **8**.

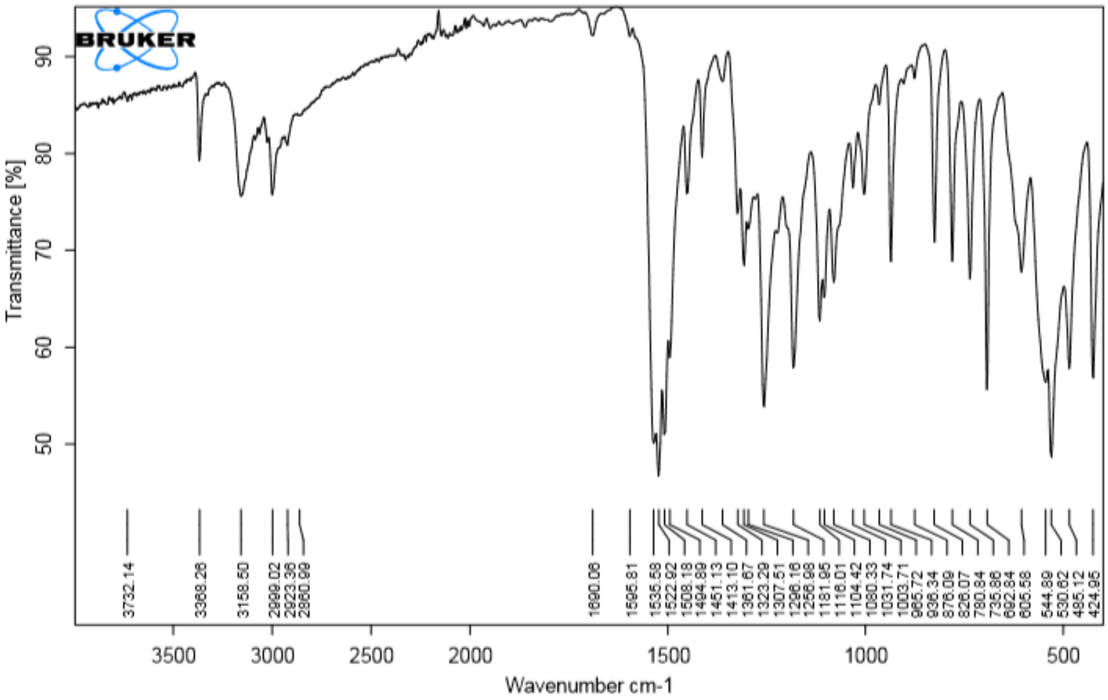

Figure S8. IR spectrum of compound **9**.

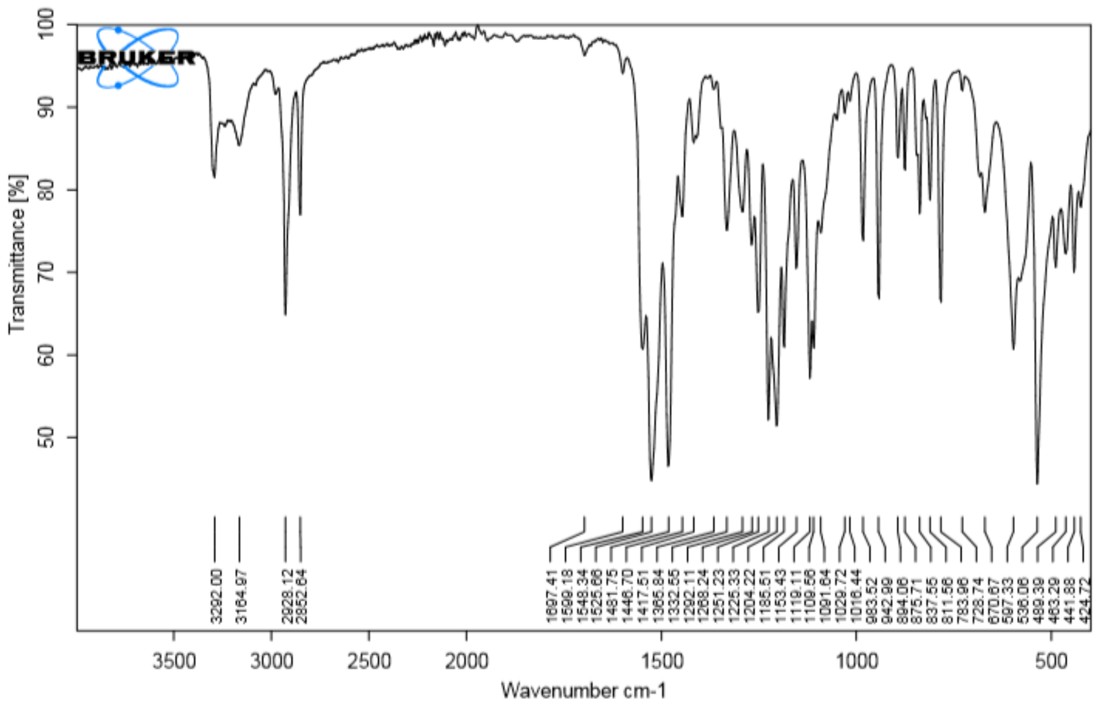

Figure S9. IR spectrum of compound **10**.

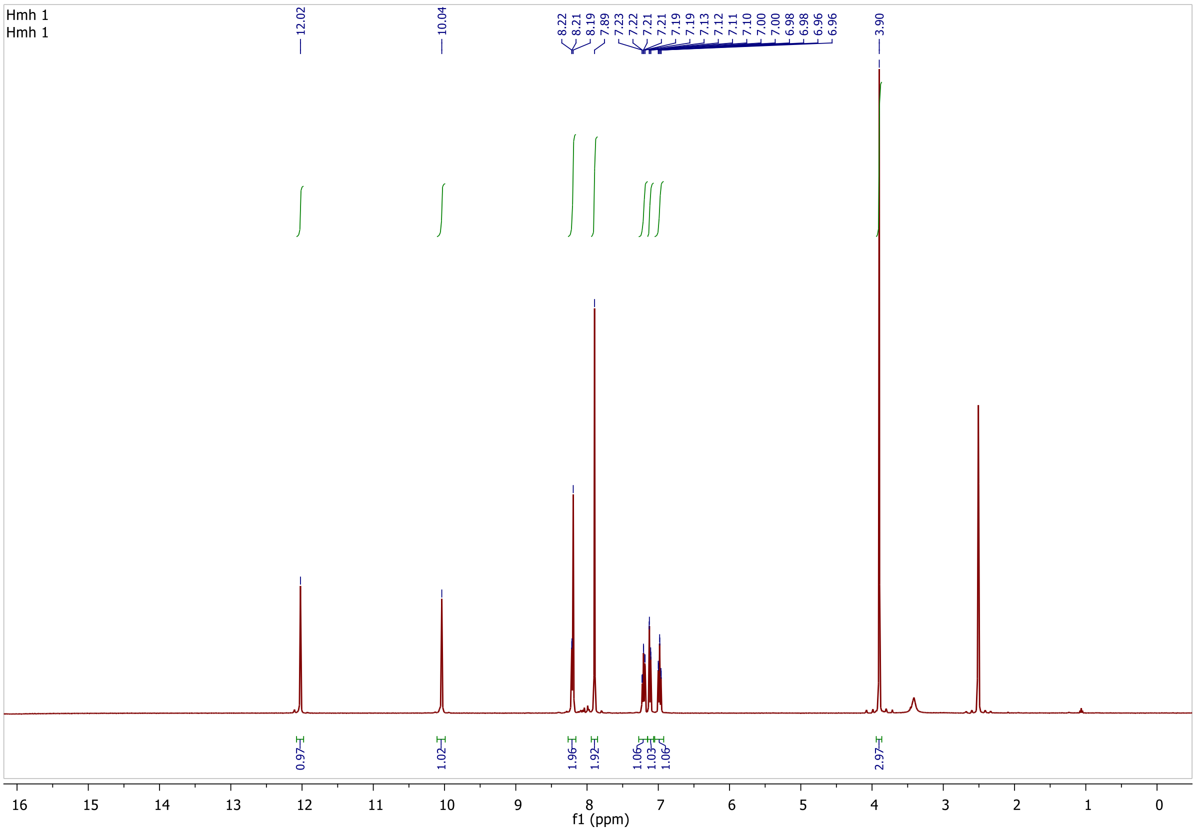

Figure S10. ^{1}H NMR spectrum of compound **1**.

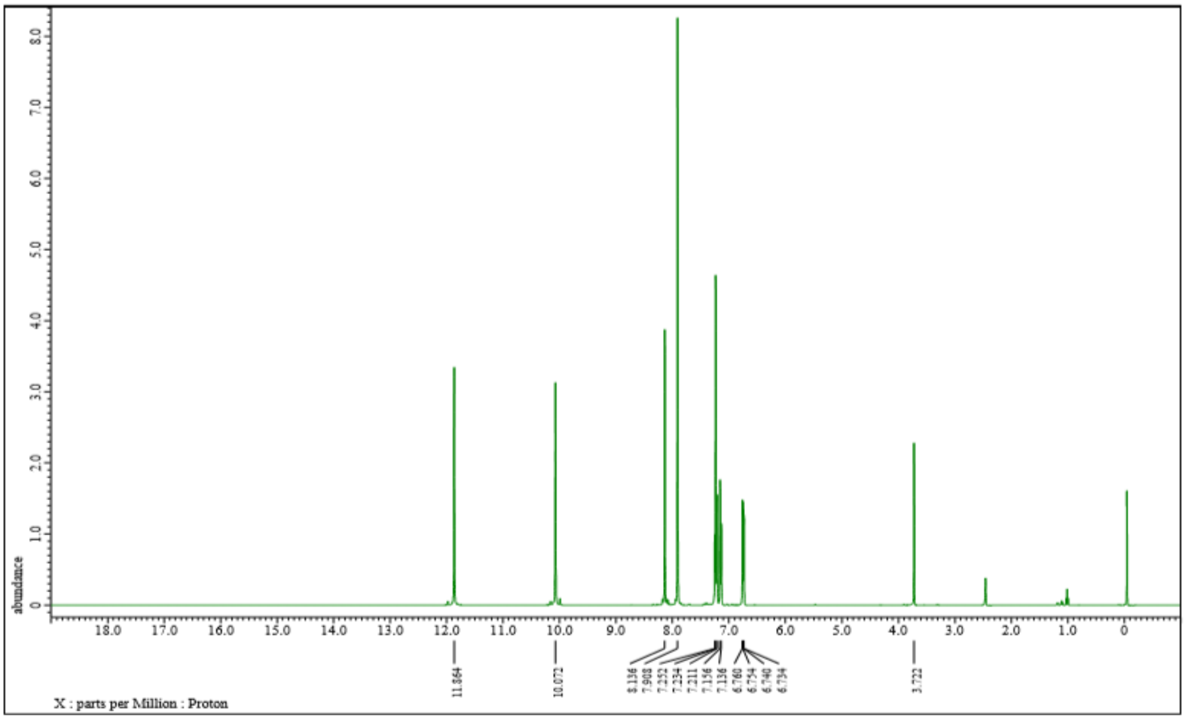

Figure S11. ^{1}H NMR spectrum of compound **2**.

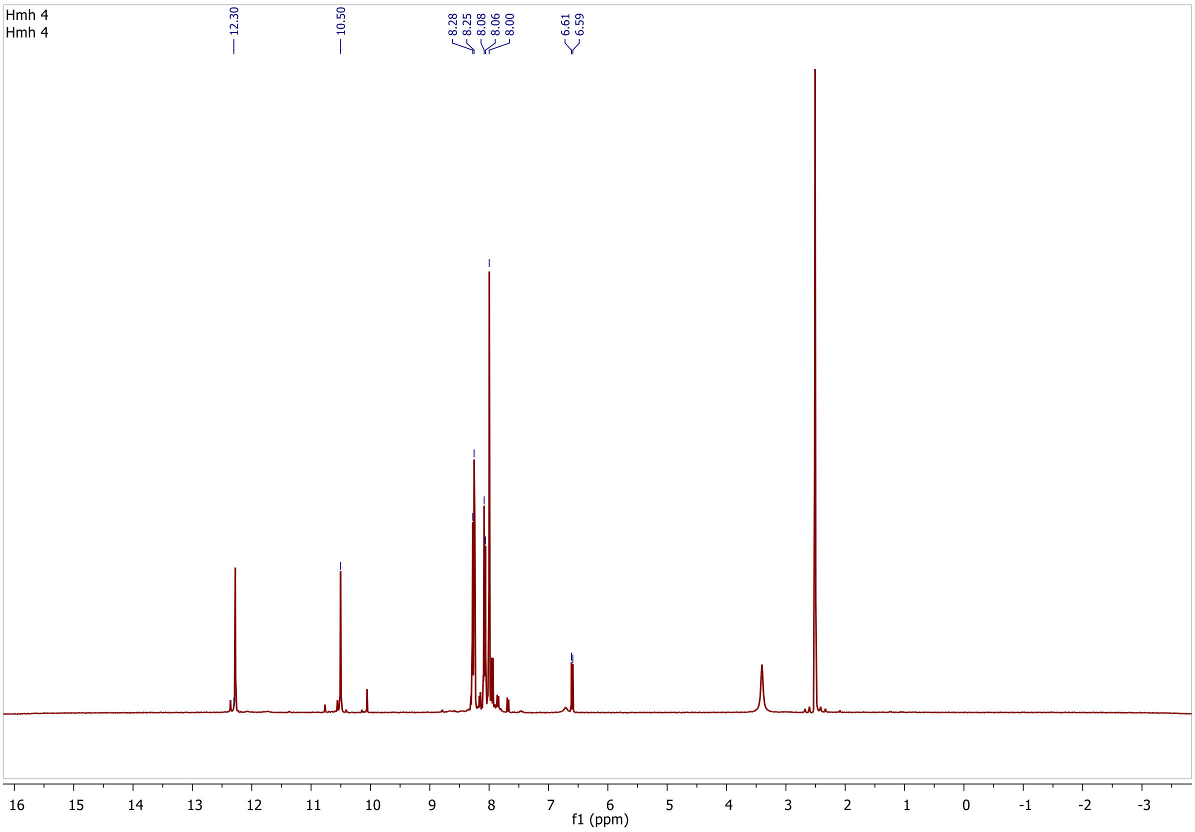

Figure S12. ^{1}H NMR spectrum of compound **3**.

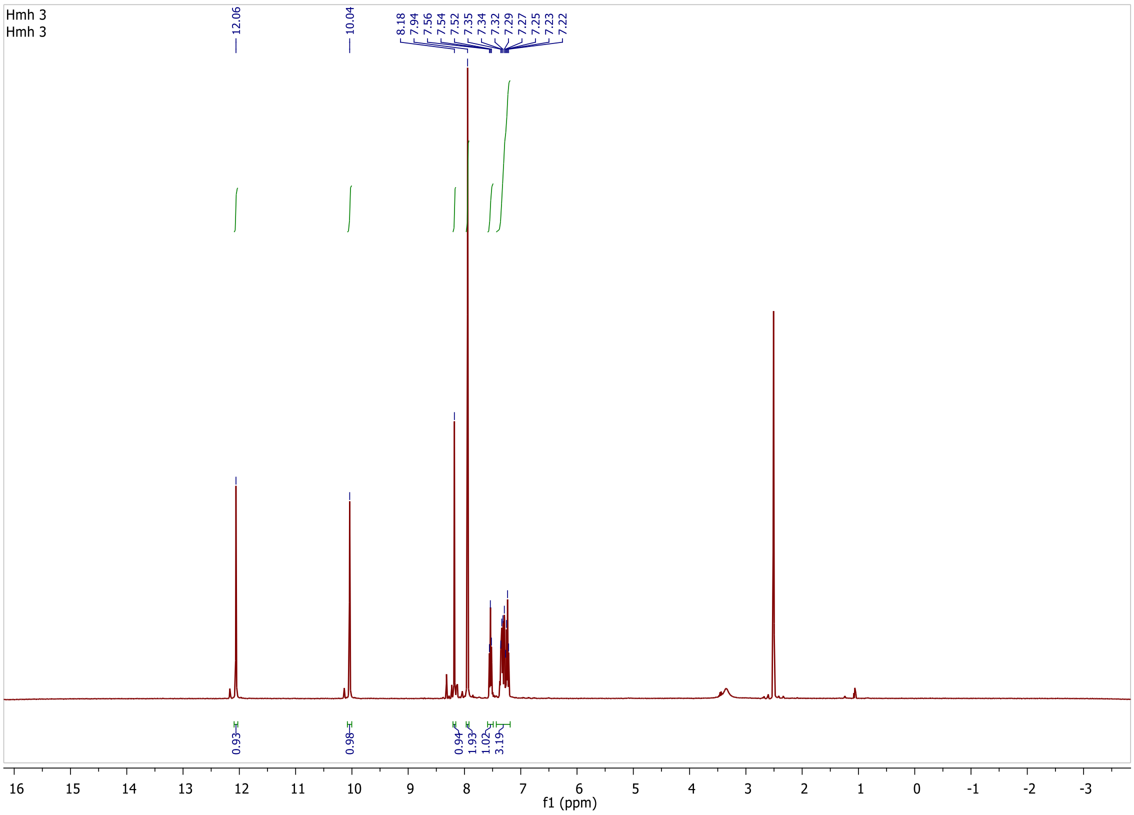

Figure S13. ^{1}H NMR spectrum of compound **5**.

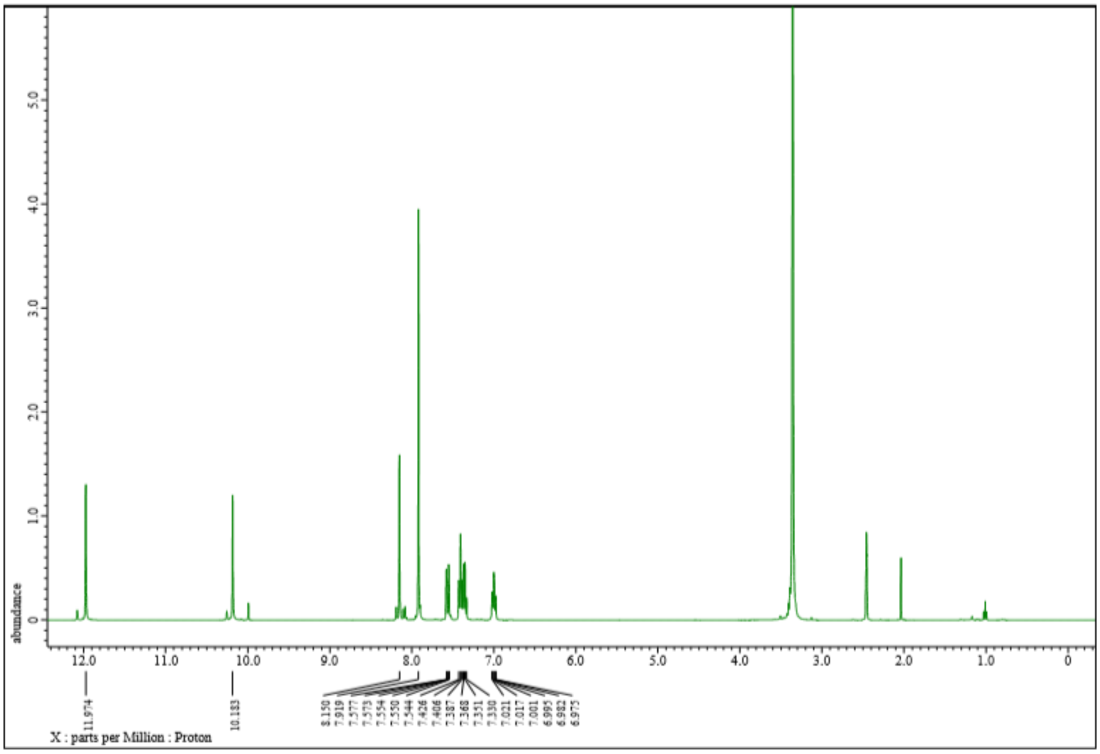

Figure S14. ^{1}H NMR spectrum of compound **6**.

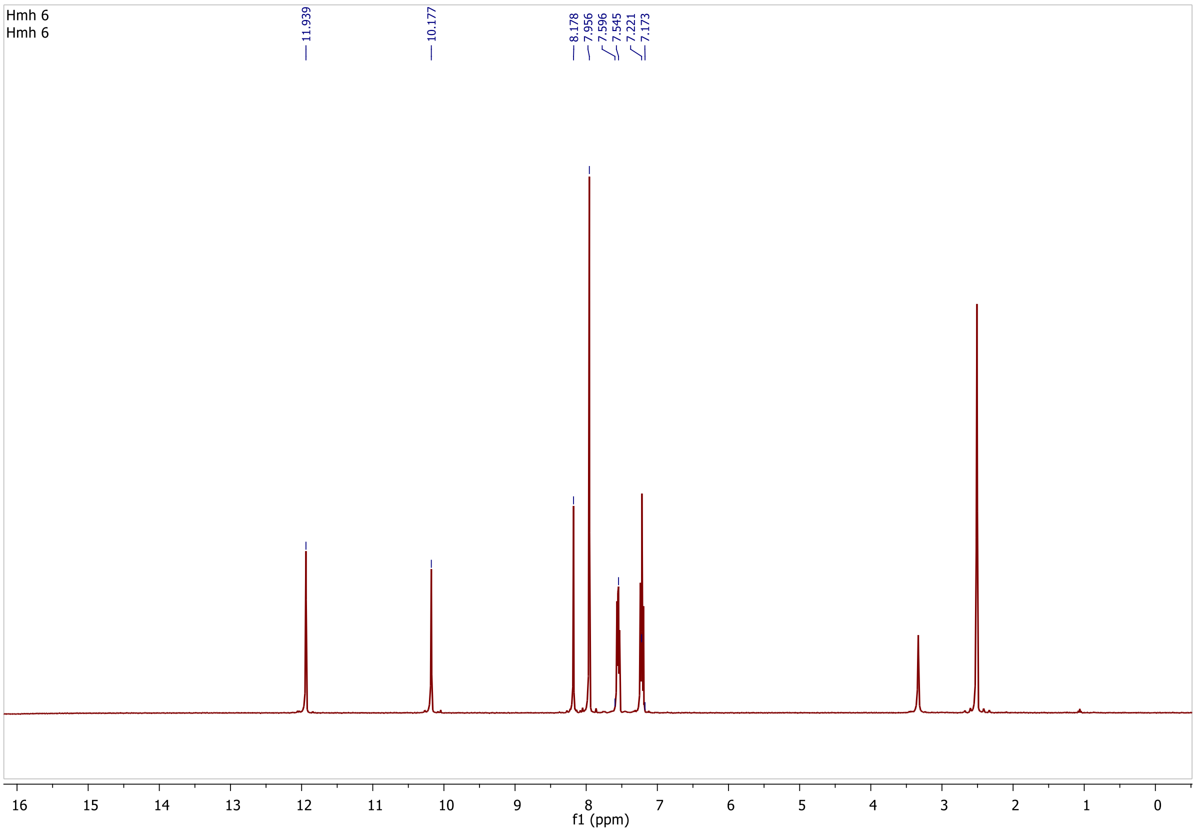

Figure S15. ^{1}H NMR spectrum of compound **7**.

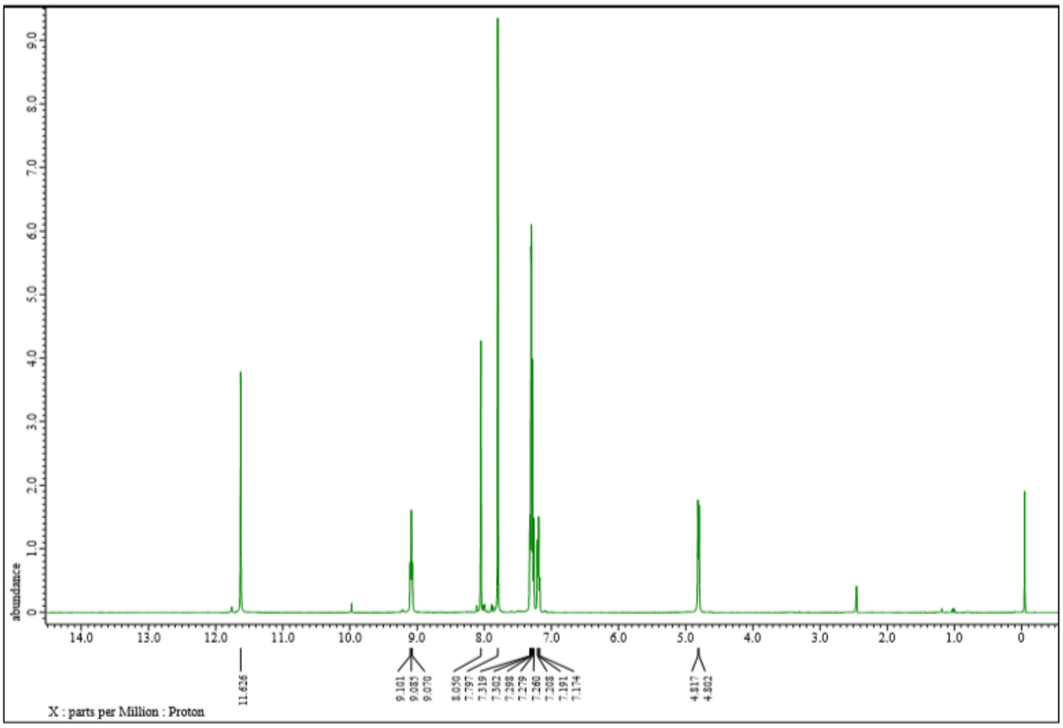

Figure S16. ^{1}H NMR spectrum of compound **8**.

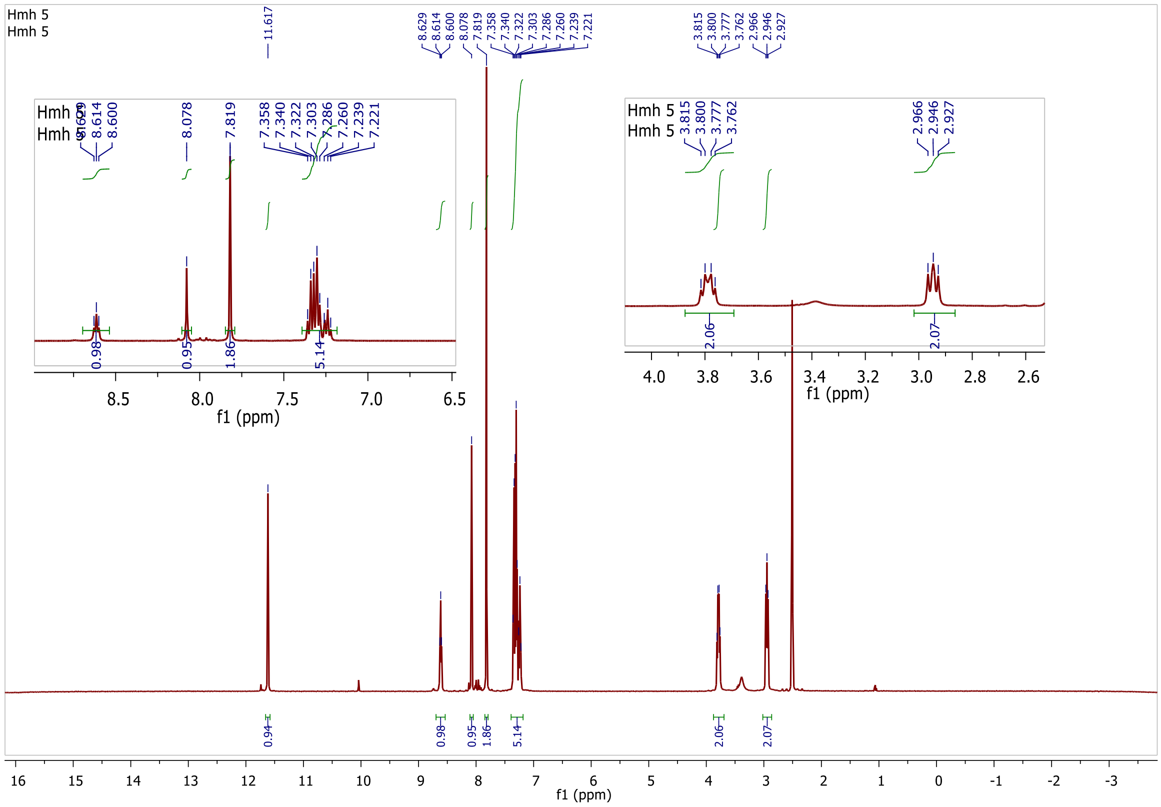

Figure S17. ^{1}H NMR spectrum of compound **9**.

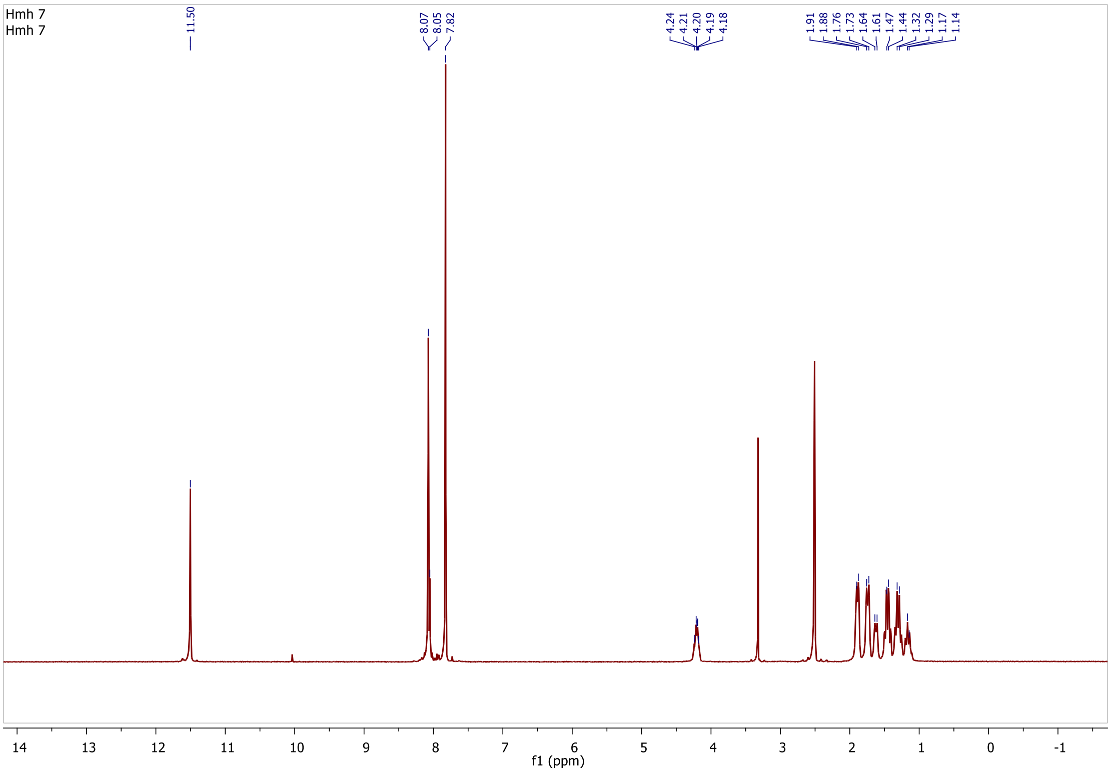

Figure S18. ^{1}H NMR spectrum of compound **10**.

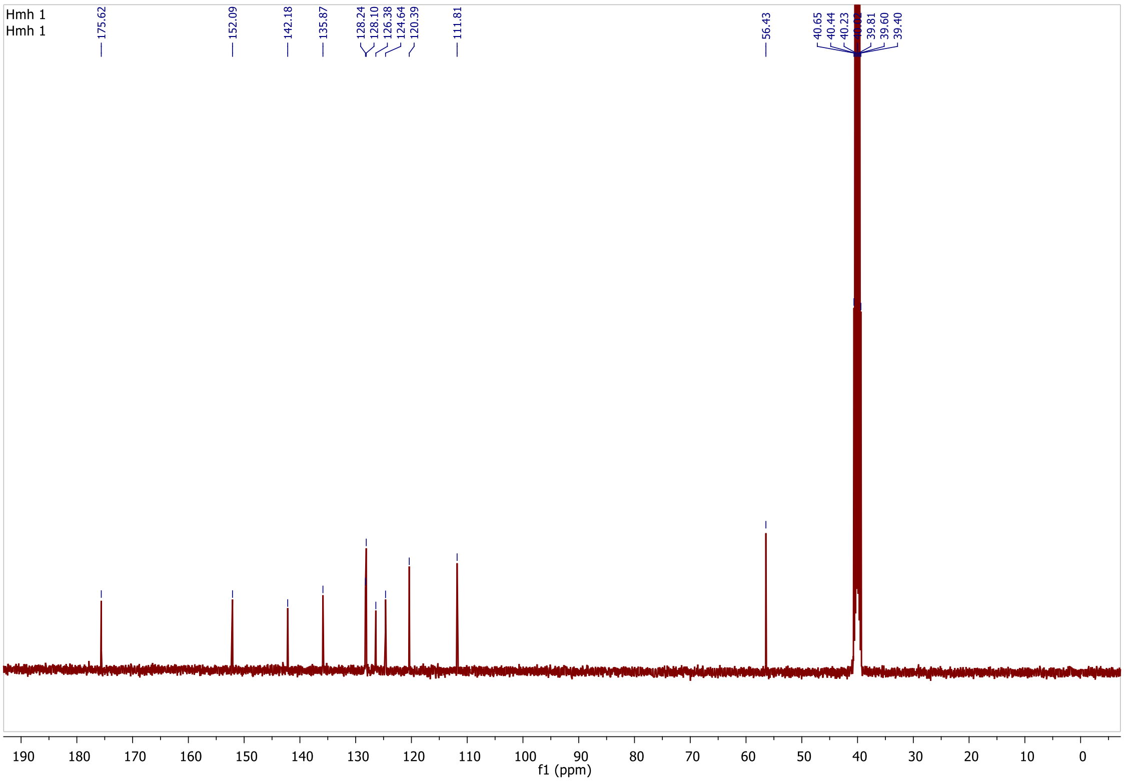

Figure S19. ^{13}C NMR spectrum of compound **1**.

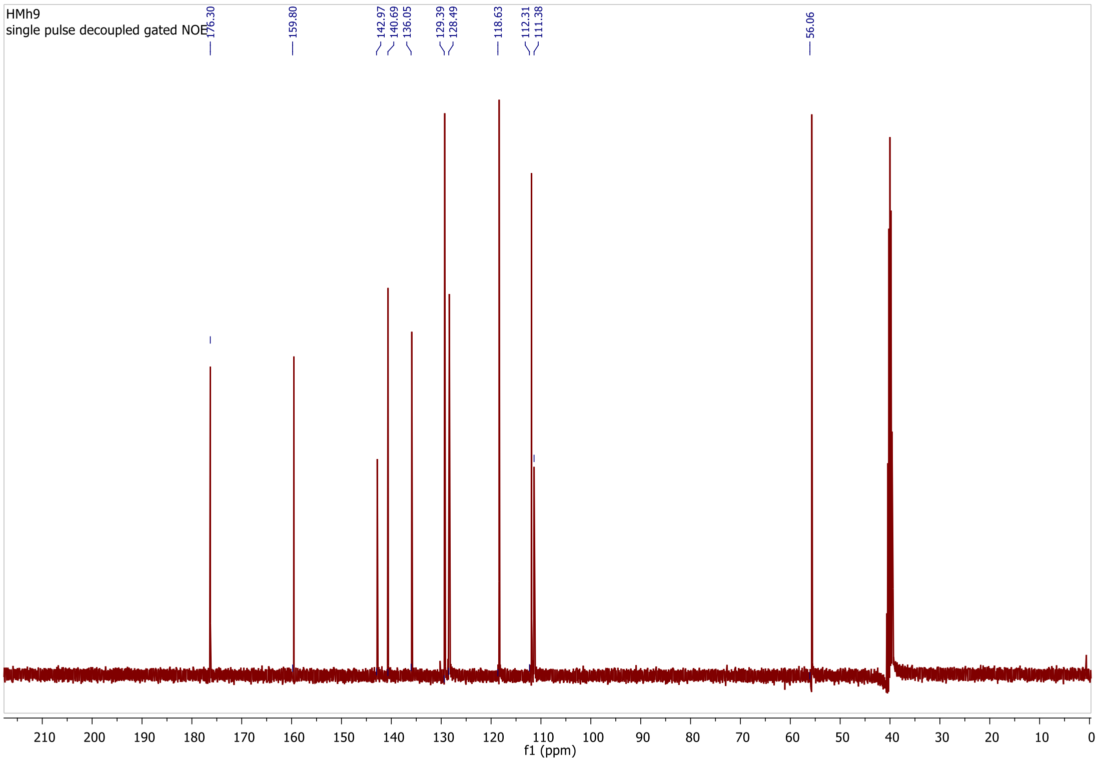

Figure S20. ^{13}C NMR spectrum of compound **2**.

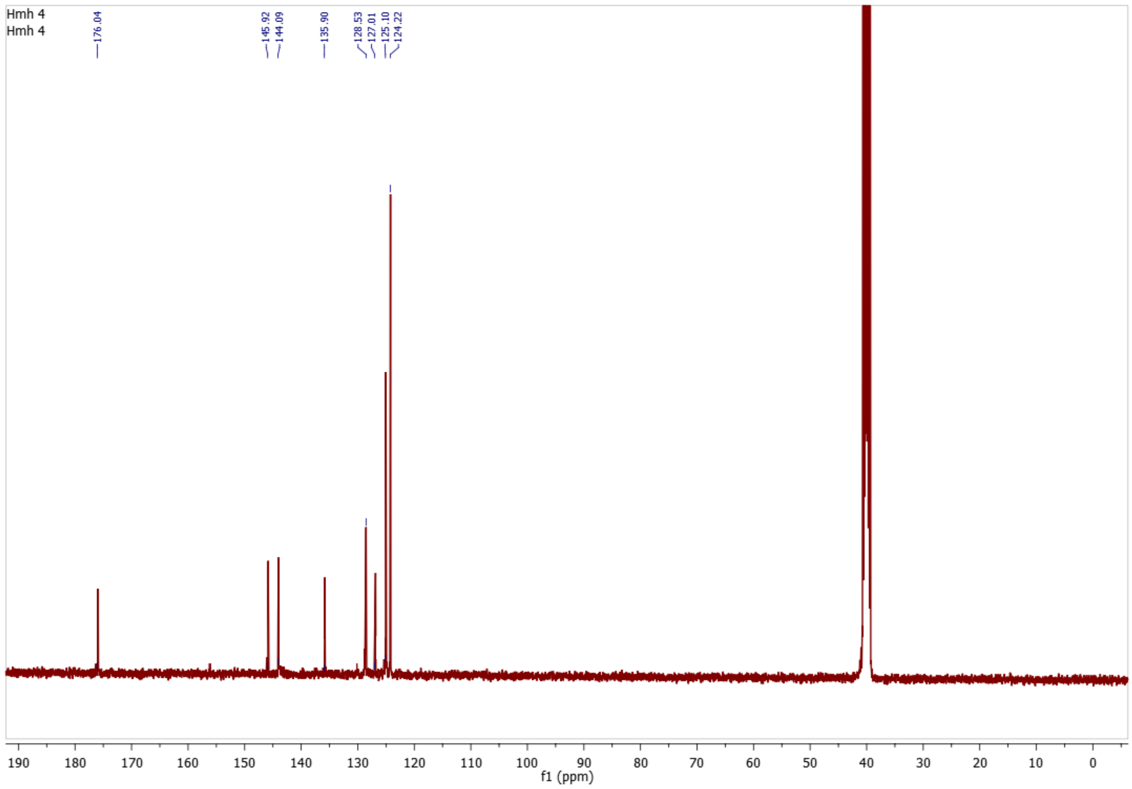

Figure S21. ^{13}C NMR spectrum of compound **3**.

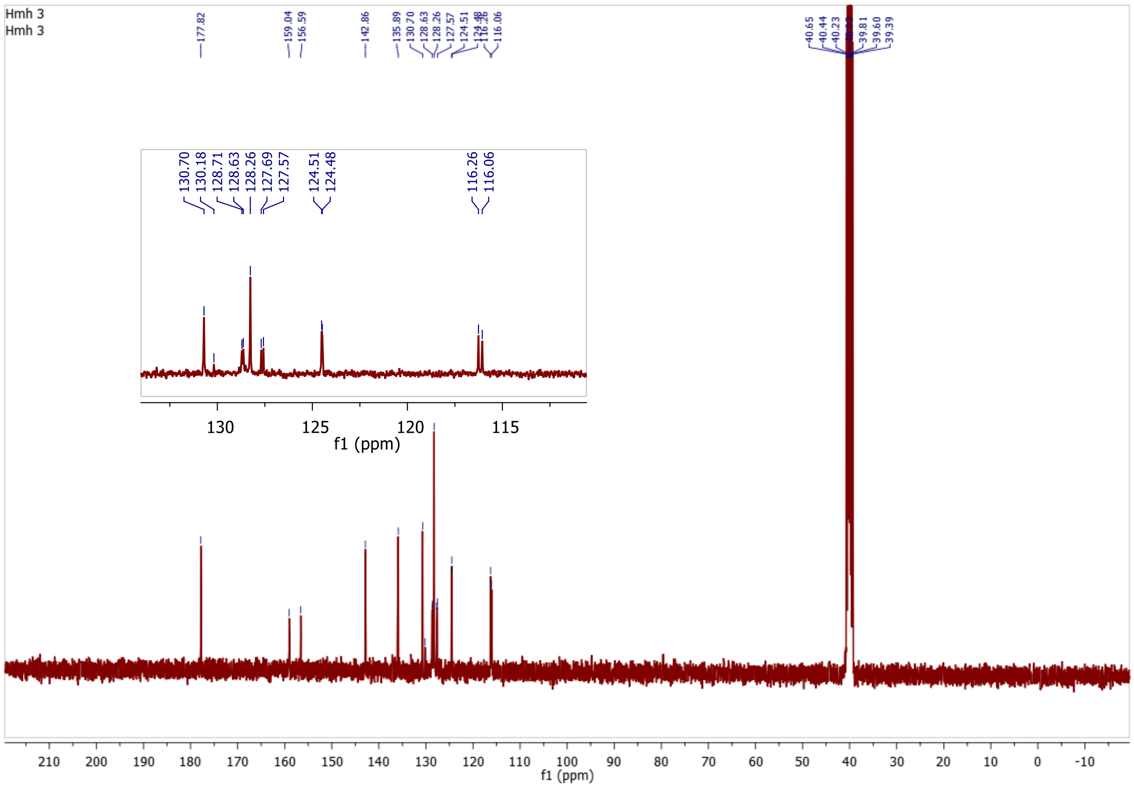

Figure S22. ^{13}C NMR spectrum of compound **5**.

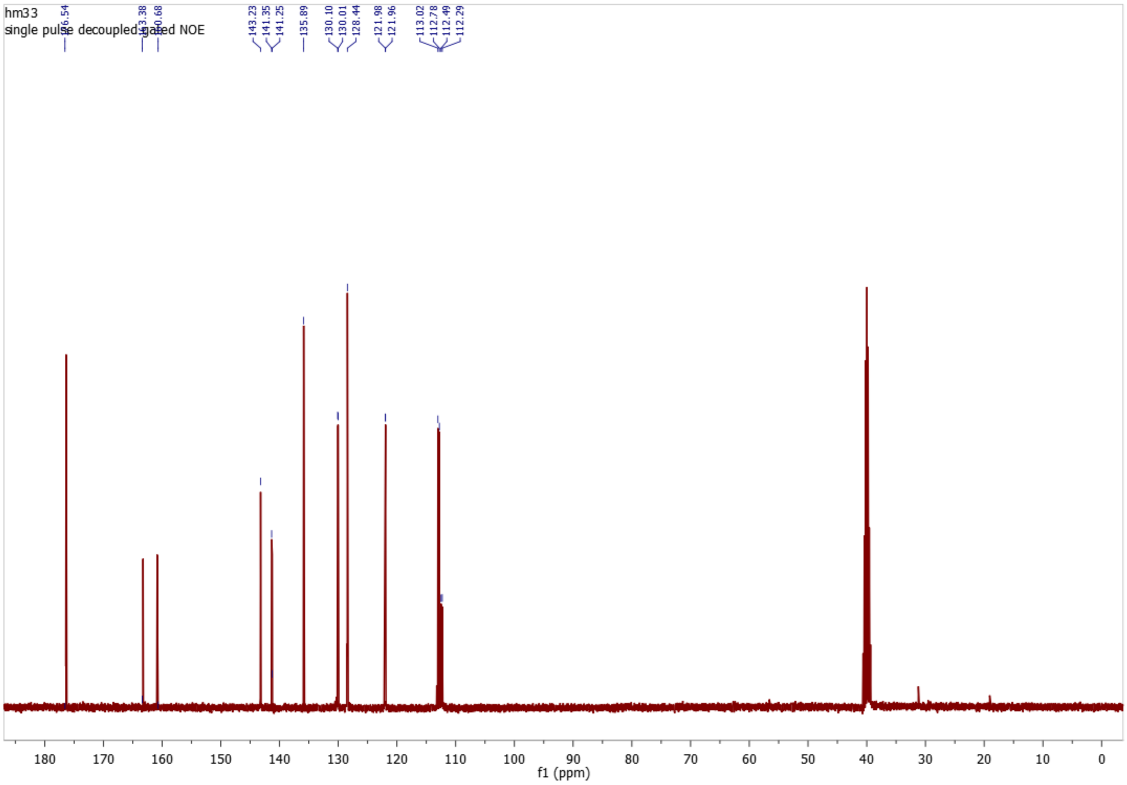

Figure S23. ^{13}C NMR spectrum of compound **6**.

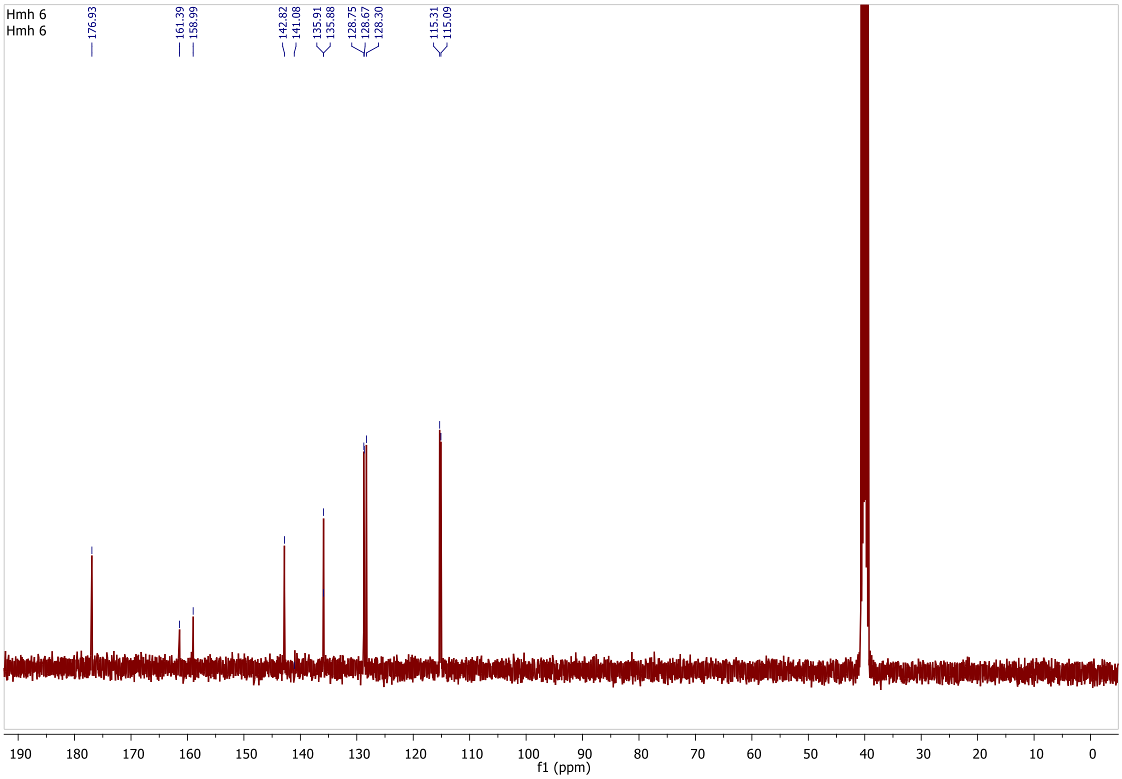

Figure S24. ^{13}C NMR spectrum of compound **7**.

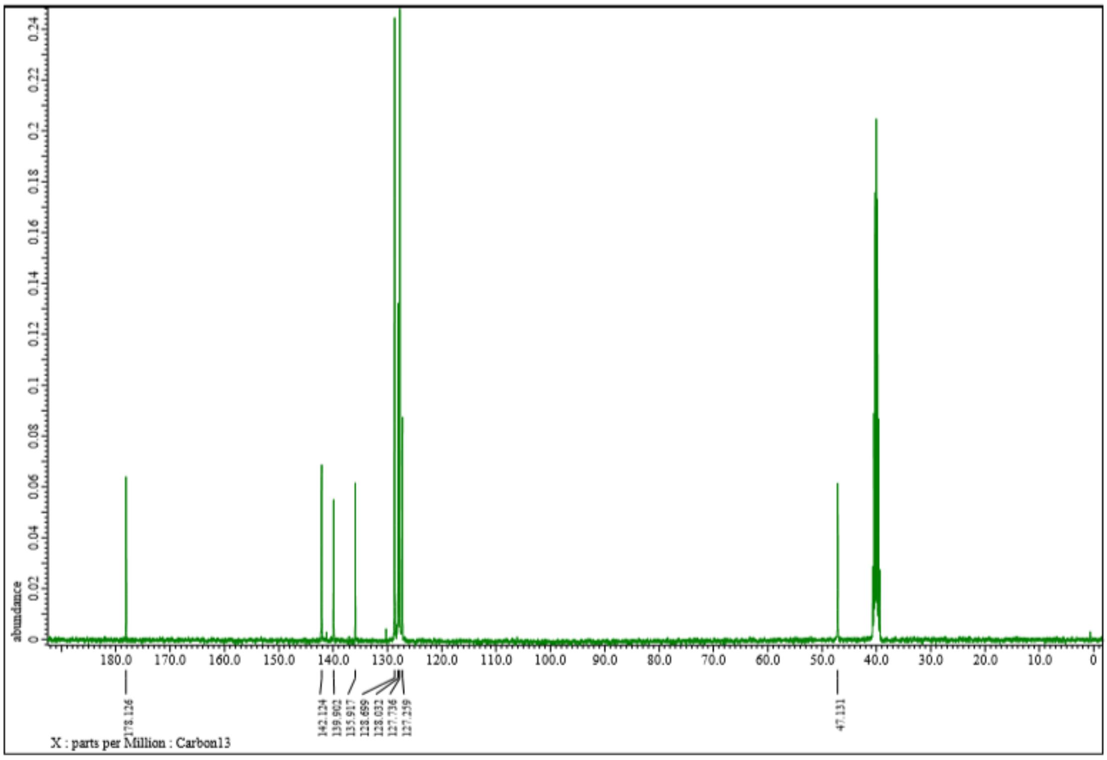

Figure S25. ^{13}C NMR spectrum of compound **8**.

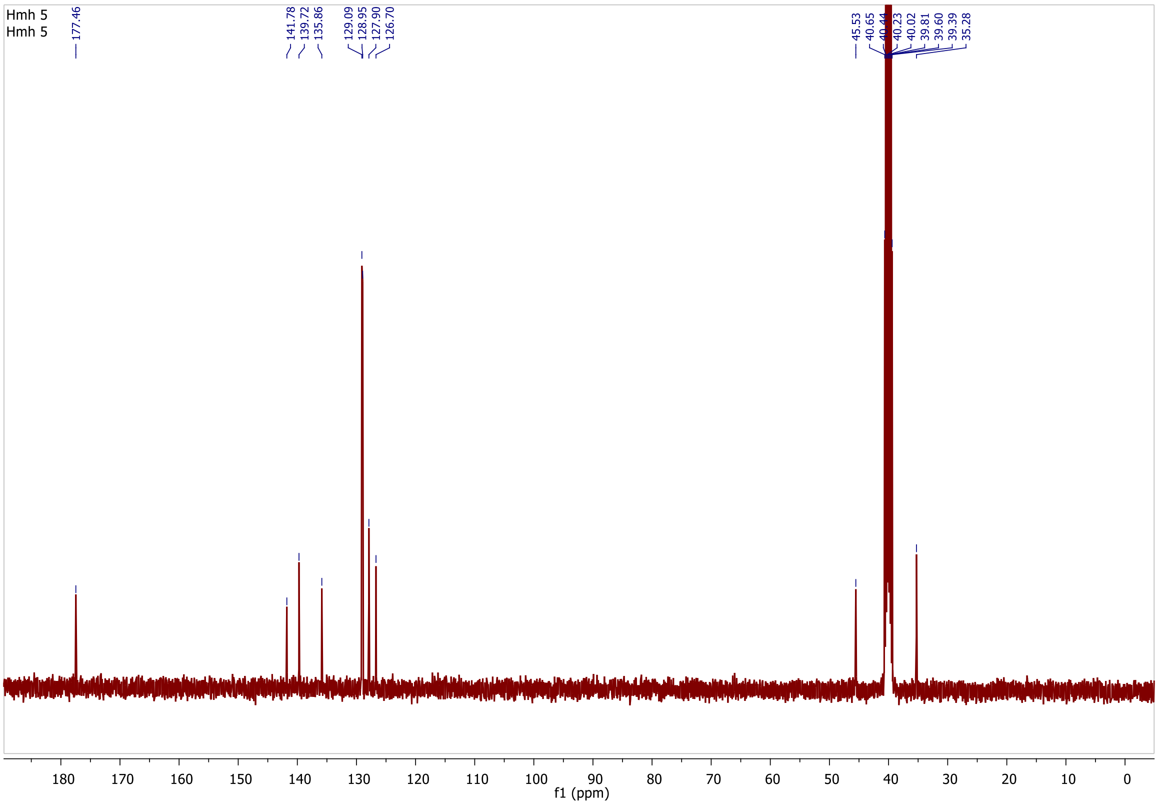

Figure S26. ^{13}C NMR spectrum of compound **9**.

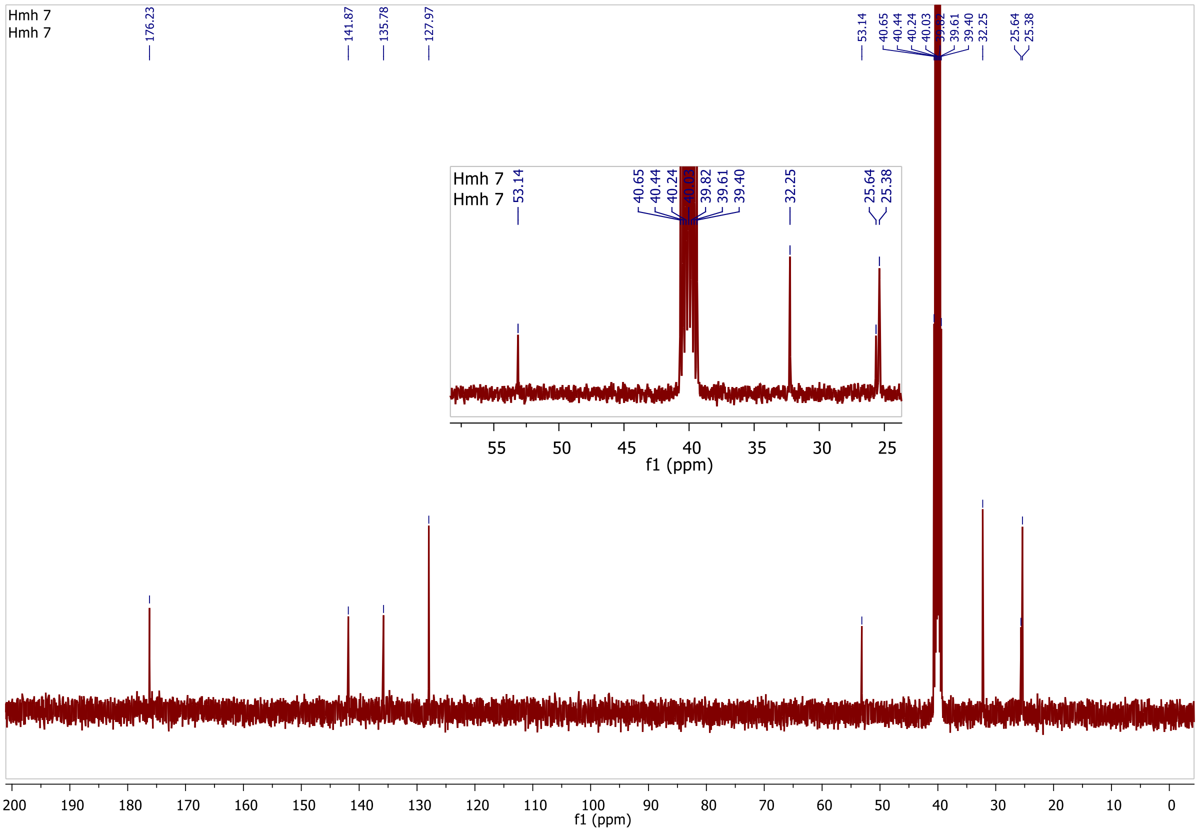

Figure S27. ^{13}C NMR spectrum of compound **10**.

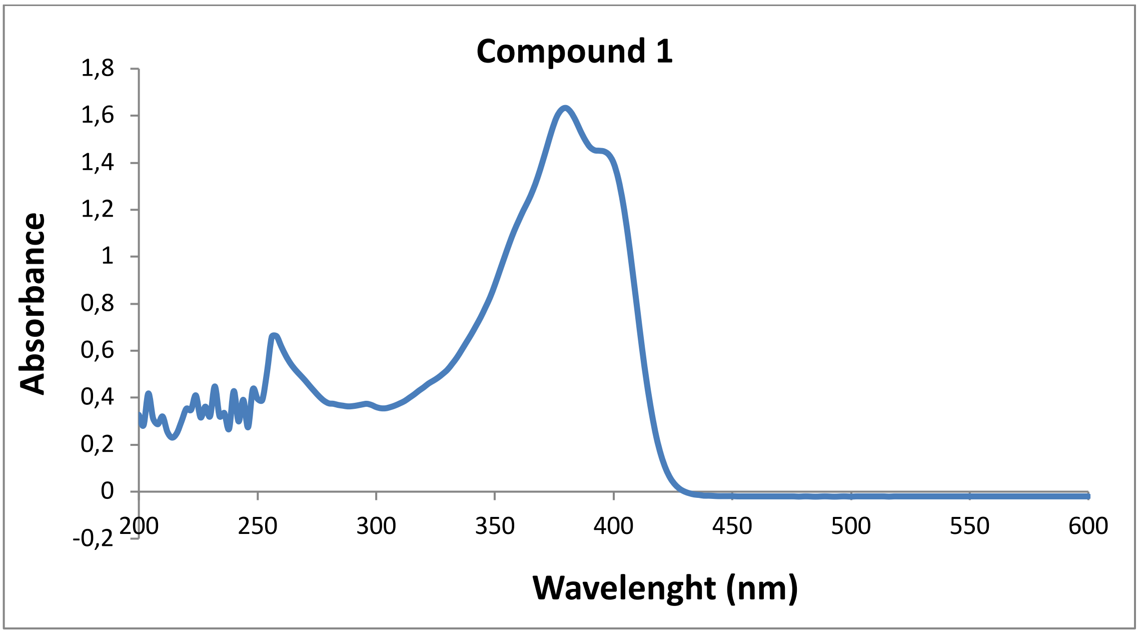

Figure S28. UV-Vis spectrum of compound **1** in DMSO.

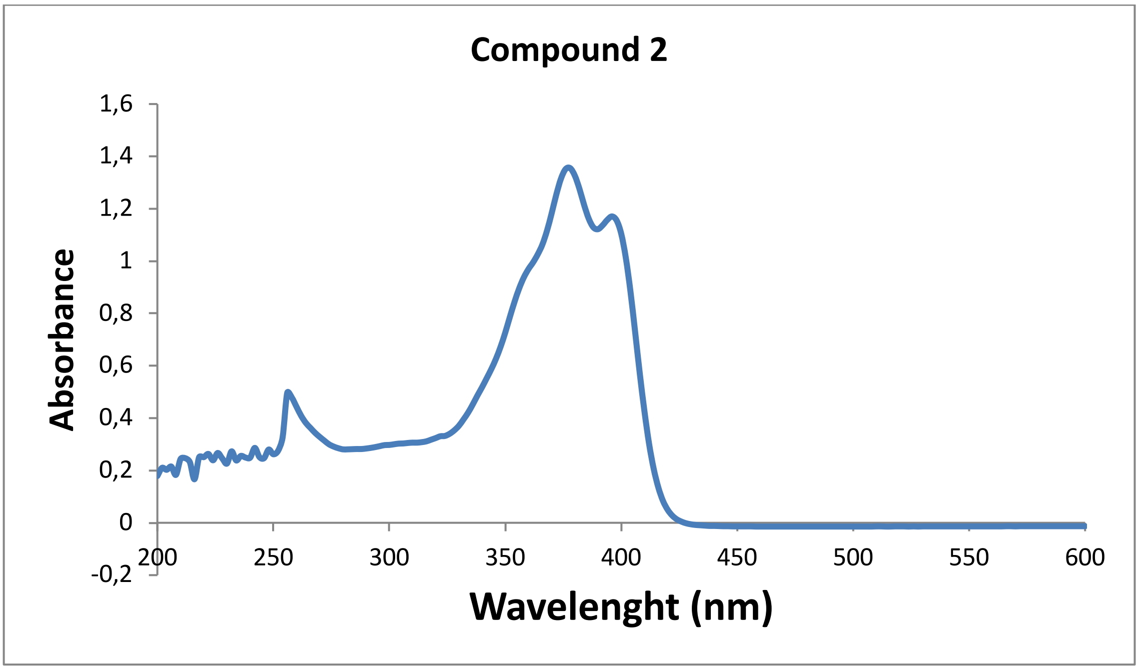

Figure S29. UV-Vis spectrum of compound **2** in DMSO.

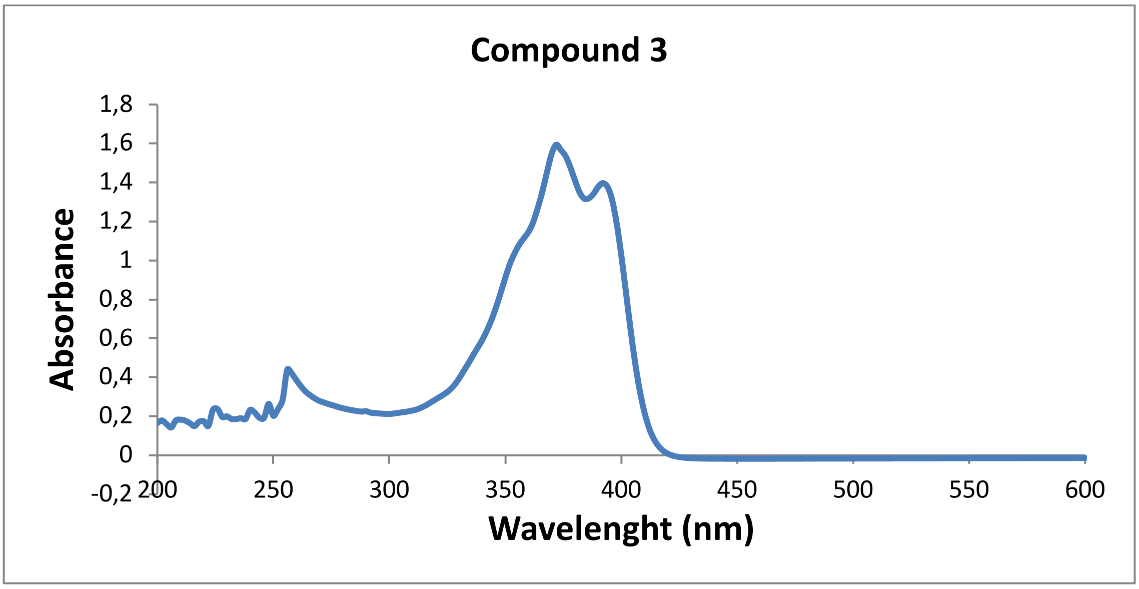

Figure S30. UV-Vis spectrum of compound **3** in DMSO.

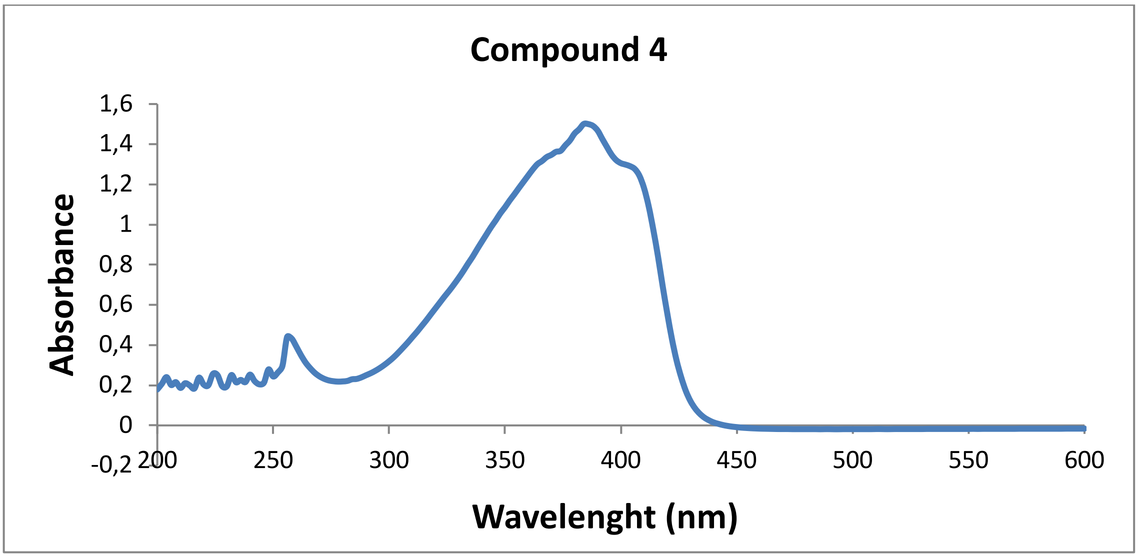

Figure S31. UV-Vis spectrum of compound **4** in DMSO.

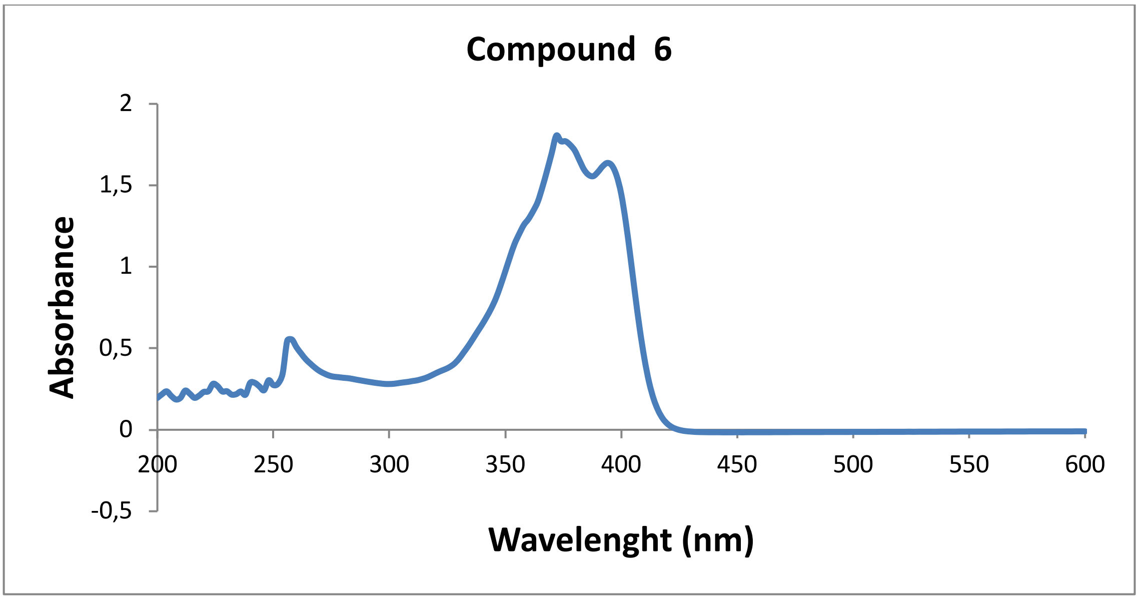

Figure S32. UV-Vis spectrum of compound **6** in DMSO.

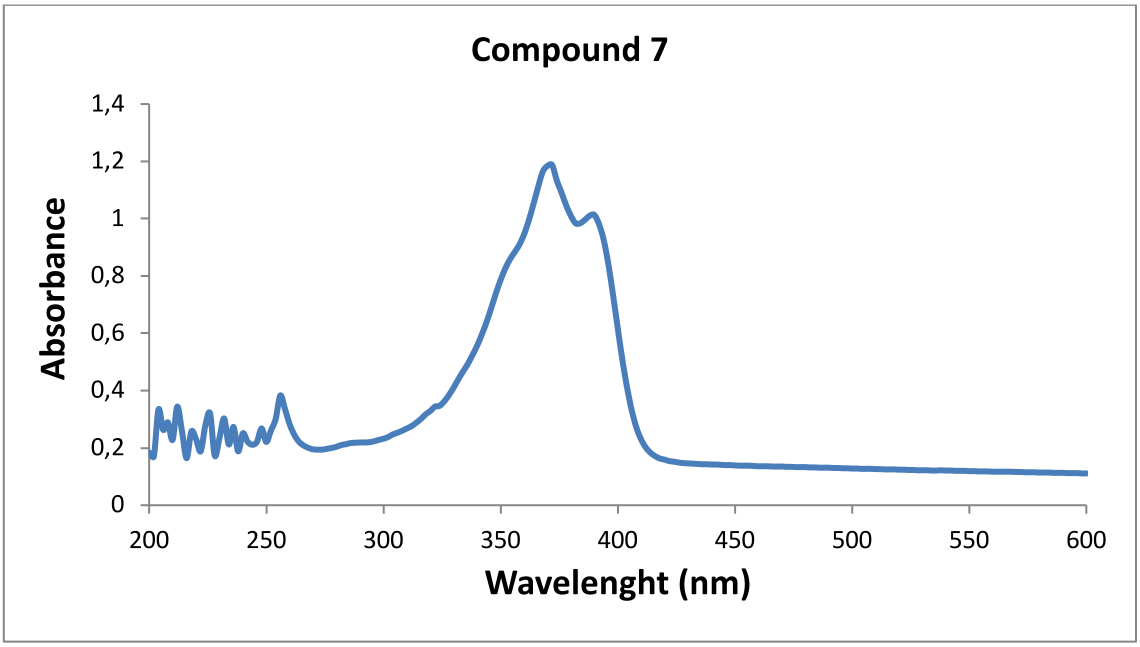

Figure S33. UV-Vis spectrum of compound **7** in DMSO.

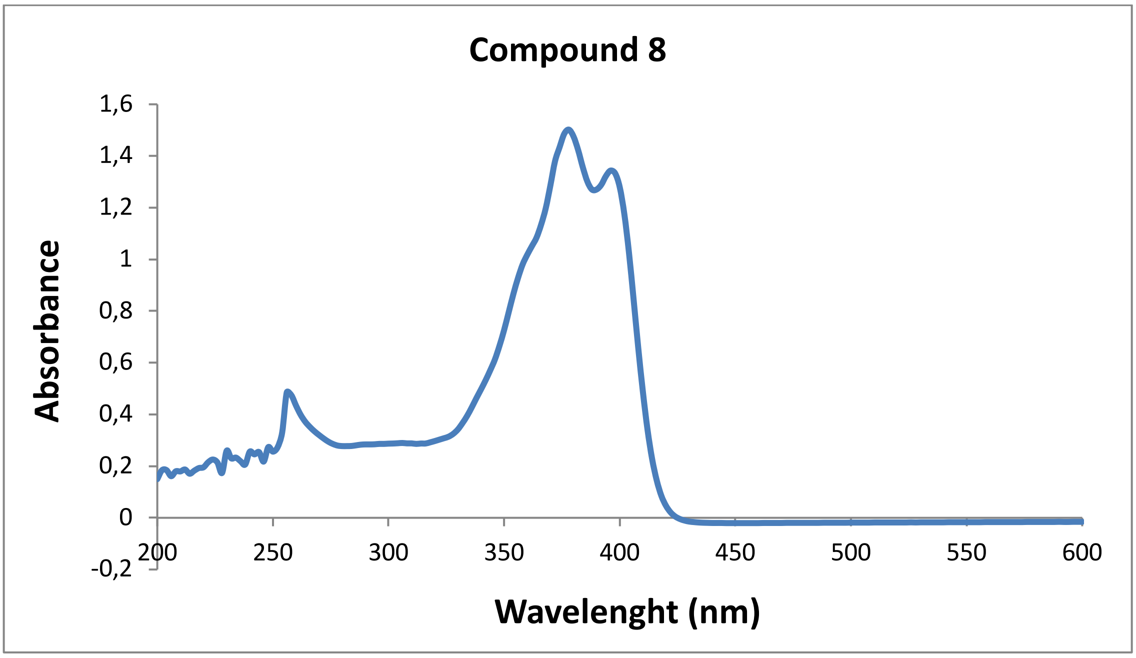

Figure S34. UV-Vis spectrum of compound **8** in DMSO.

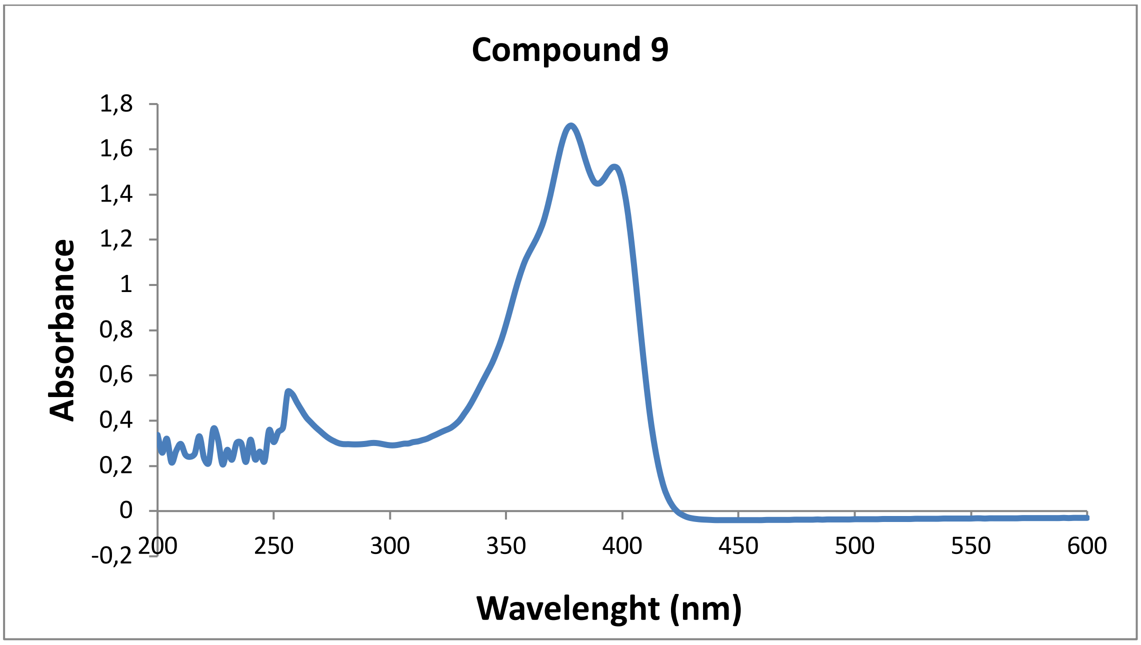

Figure S35.UV-Vis spectrum of compound **9** in DMSO.

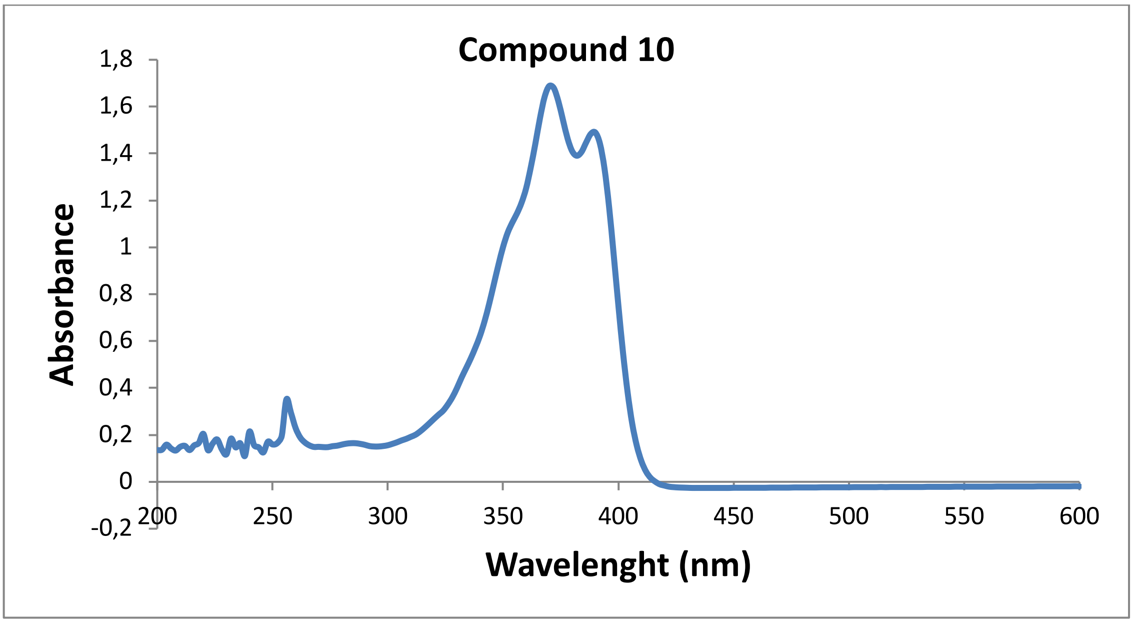

Figure S36. UV-Vis spectrum of compound **10** in DMSO.
